# Patient-reported outcome measures within European cohorts of severely injured patients: a systematic review and meta-analysis

**DOI:** 10.1007/s00068-026-03325-y

**Published:** 2026-09-04

**Authors:** Laura van den Berg, Jan C. van Ditshuizen, Pieter H. C. Leliefeld, Dennis den Hartog, Georgios F. Giannakópoulos, Michiel H. J. Verhofstad, Mariska A. C. de Jongh

**Affiliations:** 1 Netwerk Acute Zorg Brabant, Elisabeth-Tweesteden Ziekenhuis, Hilvarenbeekse Weg 60, Tilburg, 5022 GC The Netherlands; 2 Department of Surgery, Elisabeth-Tweesteden Ziekenhuis, Tilburg, The Netherlands; 3 Netwerk Acute Zorg Zuidwest, Erasmus MC, University Medical Centre Rotterdam, Rotterdam, The Netherlands; 4 Trauma Research Unit, Department of Surgery, Erasmus MC, University Medical Centre Rotterdam, Rotterdam, The Netherlands; 5 Department of Surgery & Trauma Centre/HEMS Lifeliner 1, Amsterdam UMC Location AMC, Amsterdam, The Netherlands; 6 Tranzo, Tilburg School of Social and Behavioural Sciences, Tilburg University, Tilburg, The Netherlands

**Keywords:** Severe trauma, Patient-reported outcomes, Quality of life, Psychosocial factors, Physical injury, Europe

## Abstract

**Purpose:**

Severe injury affects multiple health-related domains, yet comprehensive European data on patient-reported outcomes remain limited. This systematic review and meta-analysis evaluates patient-reported outcome measures (PROMs) use and outcomes in severely injured European cohorts.

**Methods:**

A systematic search of four databases up to October 14, 2025, identified European studies from 2000 onward reporting PROMs in severely injured patients. Severe injury was defined as an Injury Severity Score ≥ 16, Glasgow Coma Scale ≤ 8, intensive care unit admission, spinal cord injury, traumatic amputations, or pelvic fractures. Two reviewers independently screened records, with disagreements resolved by a third reviewer. Meta-analysis was performed when ≥ 3 studies reported comparable PROMs at similar follow-up timepoints.

**Results:**

Of 2,479 studies, 119 were included. Most cohorts originated from the Netherlands (26%), Norway (18%), and Germany (16%). General severely injured cohorts were most frequently studied (61%), followed by traumatic brain injury (17%), and spinal cord injury (15%). In total, 94 PROMs were used across 277 follow-up timepoints. Health-related quality of life was assessed most frequently (63%), after that anxiety/depression (14%), post-traumatic stress (9%), and social functioning (6%). At one year follow-up, the pooled EuroQol-5D-3 L index score was 0.70 (*95% CI* 0.62–0.77) and VAS score was 68 (*95% CI* 60–75), indicating persistent impairment compared to population norms.

**Conclusion:**

Severely injured patients show persistent impairments with incomplete restoration of pre-injury functioning. Despite increased PROMs use, heterogeneity in selection and outcome reporting limits comparability, underscoring the need for standardised PROM assessment to improve outcome evaluation after severe injury.

**Supplementary Information:**

The online version contains supplementary material available at https://doi.org/10.1007/s00068-026-03325-y.

## Introduction

Severe traumatic injury represents a major global challenge. Trauma is accountable for substantial morbidity and is one of the leading causes of death, especially in patients aged 50 years or younger [1, 2]. With improvements in trauma care, survival rates have increased during the last decades [3–5]. Consequently, a growing population of trauma survivors is living with long-lasting sequelae. These long-term consequences frequently include persistently physical limitations, chronic pain, psychological distress, and reduced social participation [6, 7]. This underlines the need for comprehensive and long-term assessment of health-related quality of life (HRQoL), allowing for early identification of persistent complaints and enabling healthcare professionals to tailor interventions. Furthermore, it offers patients clearer insights into their recovery trajectories, which can facilitate informed decision-making and encourage active participation in the rehabilitation process.

Several factors influence HRQoL following severe traumatic injury. Injury severity, pre-existing health conditions, social and psychological factors, and personality traits all play a role in shaping recovery outcomes [6, 8–10]. Furthermore, severely injured patients are more likely to face mental health challenges that are associated with poorer HRQoL outcomes [8, 11–13]. Psychological consequences are often under-recognised yet contribute significantly to a diminished HRQoL. The interaction between persistent physical disability and psychological distress may result in long-lasting limitations in daily functioning and difficulties to normal life for years post-trauma [14].

Patient-reported outcome measures (PROMs) provide a structured method to assess patients’ perceptions of health status and functioning. PROMs can support clinical decision-making, rehabilitation planning, and evaluation of quality of care, thereby contributing to improved short- and long-term outcomes for trauma survivors [7]. PROMs can be generic or disease specific. Generic PROMs, such as EuroQol-5D (EQ-5D) and 36-Item Short Form Health Survey (SF-36), are applicable across diseases and a wide range of patient populations. Generic PROMs can capture one or multiple health domains, including physical functioning, pain, mental health, and social participation [15–17]. Disease specific PROMs, for example the Quality of Life After Brain Injury Questionnaire (QOLIBRI) and the Lower Extremity Function Scale (LEFS) [18, 19], are tailored to specific patient groups or injury patterns. Disease specific PROMs provide detailed insight into condition-specific health complaints and functional limitations.

Given the international diversity in trauma care and treatment strategies, standardised generic PROMs are required to facilitate and evaluate quality and effectiveness of care in different healthcare systems. Analysing patient subgroups remains challenging since most studies include relatively small cohorts and often report low response rates, limiting robustness of subgroup analyses.

Pooling data across cohorts enables more robust conclusions and offers insight into the development and current state of PROMs use in European trauma care. This systematic review and meta-analysis provides an overview of PROMs used in European cohorts of severely injured patients in the 21st century.

## Methods

### Search strategy

A systematic literature search was conducted across four electronic databases, including Medline AA, Embase, Web of Science Core Correlation, and CINAHL Plus. The search was performed in accordance with Preferred Reporting Items for Systematic Reviews and Meta-Analysis (PRISMA) guidelines and registered in PROSPERO (CRD420251010104). A combination of relevant keywords and MeSH terms related to trauma, severe injury, PROMs, and European countries was used. All identified records were imported into Covidence© systematic review software to facilitate screening. Two reviewers independently screened titles and abstracts for eligibility. Full-text assessment was performed selectively for studies considered potentially eligible. Discrepancies were resolved by consultation with a third reviewer. The final search was completed on October 14, 2025.

### Eligibility criteria

Studies were eligible if they (1) were conducted in Europe, (2) were published from 2000 onwards, and (3) included cohorts of severely injured patients. Severe injury was defined as injury severity score (ISS) ≥ 16, traumatic brain injury (TBI) with a Glasgow coma scale (GCS) ≤ 8, intensive care unit (ICU) admission, spinal cord injury (SCI), traumatic limb amputations, severe burns, or severe pelvic fractures. Studies were excluded if they did not assess PROMs, focused on non-traumatic or combat-related injuries, or if the full text was unavailable.

### Data extraction

Data extraction was performed using a standardised form in Covidence©. Extracted data included study characteristics (author, year of publication, country, study design, sample size), patient demographics, trauma subgroup, PROMs used, and quantitative study results. Trauma subgroups were categorised as general severely injured populations, defined as cohorts of patients with severe injury but not classified into specific subgroups, TBI, SCI, burns, pelvic fractures, or paediatric populations. In addition, the type and frequency of PROMs used across studies were assessed. All PROMs were systematically classified into three overarching health domains including (1) HRQoL, (2) psychological outcomes including anxiety, depression, and post-traumatic stress, and (3) social participation.

### Quality assessment

Methodological quality and risk of bias were assessed for each included study using a predefined checklist developed by the review team and implemented in Covidence©. Since this review focused on the methodological quality and reporting of PROM-based observational cohort studies, rather than on the effect of an intervention or exposure, a review-specific checklist was considered most appropriate. The checklist was informed by domains included in established quality assessment and risk-of-bias instruments for observational studies and tailored to the objectives of this review. Assessed domains included completeness of outcome reporting, clarity of population definition, response rates, potential recall bias, reporting physical and psychiatric comorbidities, and data collection methods.

### Data analysis and synthesis

Descriptive analyses were performed to characterise the included studies. The number of studies was summarised per country and per publication year to assess geographical distribution and temporal trends in PROMs use. Studies were additionally categorised by trauma populations to describe patterns of PROM application across subgroup cohorts. Since patient-reported outcomes change over time after injury, follow-up duration was considered an important source of heterogeneity. Therefore, PROM results were synthesised according to comparable follow-up timepoints whenever possible, and a meta-analysis was only performed when at least three studies reported the same PROM at similar follow-up intervals.

For studies reporting the EQ-5D without specifying the instrument version (3–5 L), predefined decision rules were applied. Studies with a study period entirely before 2009 were assumed to have used the EQ-5D-3 L, as the EQ-5D-5 L was introduced in 2009 [20]. For studies conducted after 2009 in which the EQ-5D version was not specified, corresponding authors were contacted for clarification. In the absence of a response, the EQ-5D version was classified as EQ-5D-not further specified (EQ-5D-NFS).

For each health domain, outcomes were synthesised using a structured approach. Continuous variables were reported as means with standard deviations (SD) or medians with interquartile ranges (IQR). When studies reported median values, mean values and corresponding SDs were estimated when feasible. If not, additional data were requested from the original authors. Where available, absolute numbers and percentages were extracted to assess the proportion of patients reporting specific outcomes. For the most frequently used PROMs, outcomes trajectories over time were visualised using scatterplots.

Random-effects meta-analyses were conducted to estimate pooled effect sizes, expressed as pooled mean differences or standardised mean differences with corresponding 95%-confidence intervals (95% CI). Statistical heterogeneity was assessed using the *I*^*2*^-statistic and visual inspection of forest plots.

All statistical analyses were conducted using IBM SPSS Statistics version 30 (IBM Corp. Armonk, NY, USA) and RStudio version 2023.12.0 (Posit Software, PBC, Boston, MA, USA). Statistical significance was defined as a *p*-value < 0.05.

## Results

### Literature search

**Fig. 1:**
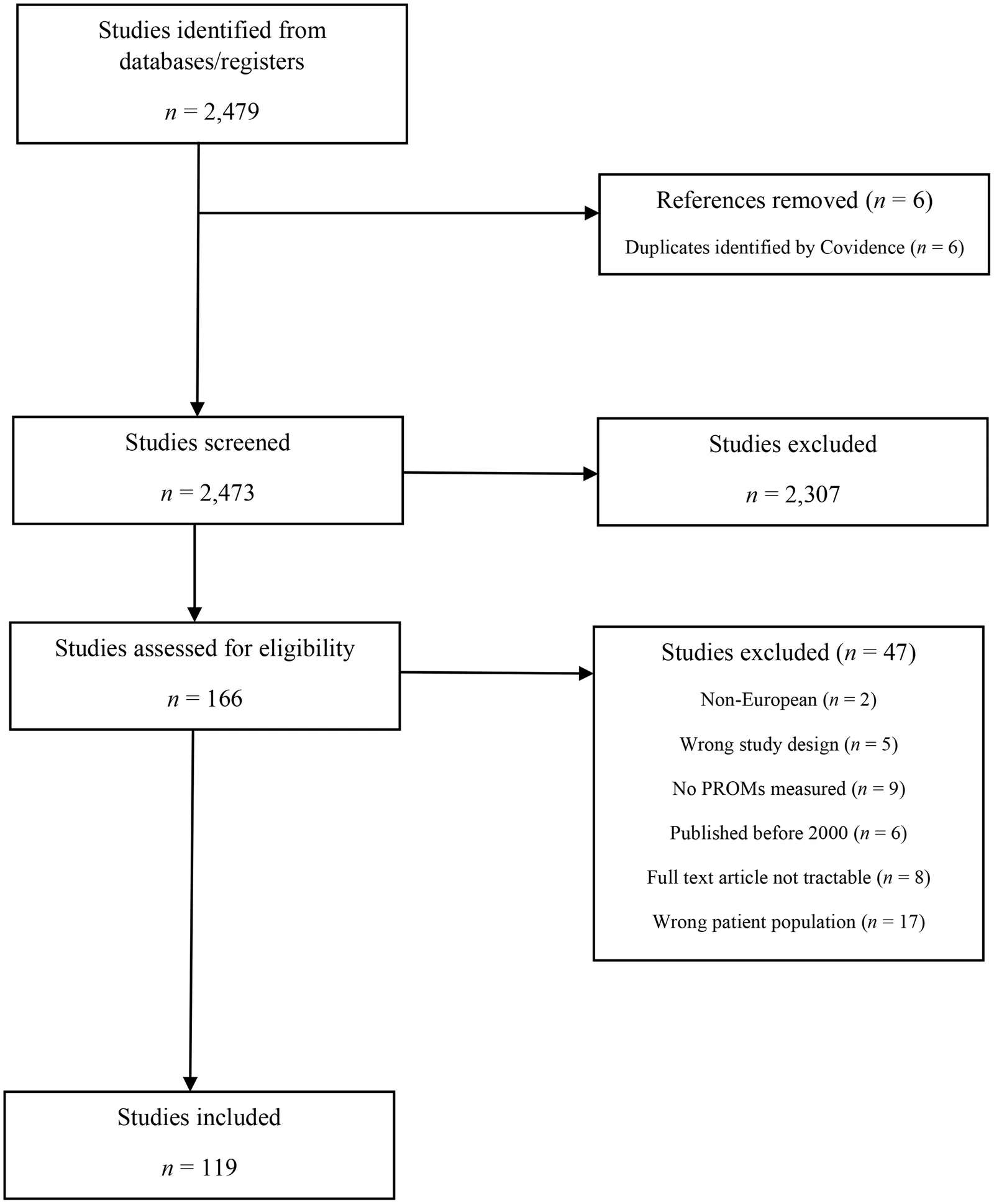
PRISMA flowchart of study inclusions

The search identified 2,479 potentially relevant studies. After removing 6 duplicates, 2,473 studies were screened on title and abstract. This screening resulted in exclusion of 2,307 studies, leaving 166 full-text studies to check eligibility for inclusion. A total of 119 studies were included in final analysis. Main reasons for exclusion were non-traumatic patient cohorts, non-tractable full text articles, and no usage of PROMs. A flow diagram according to PRISMA guidelines is shown in Fig. 1.

### Baseline study characteristics

**Fig. 2:**
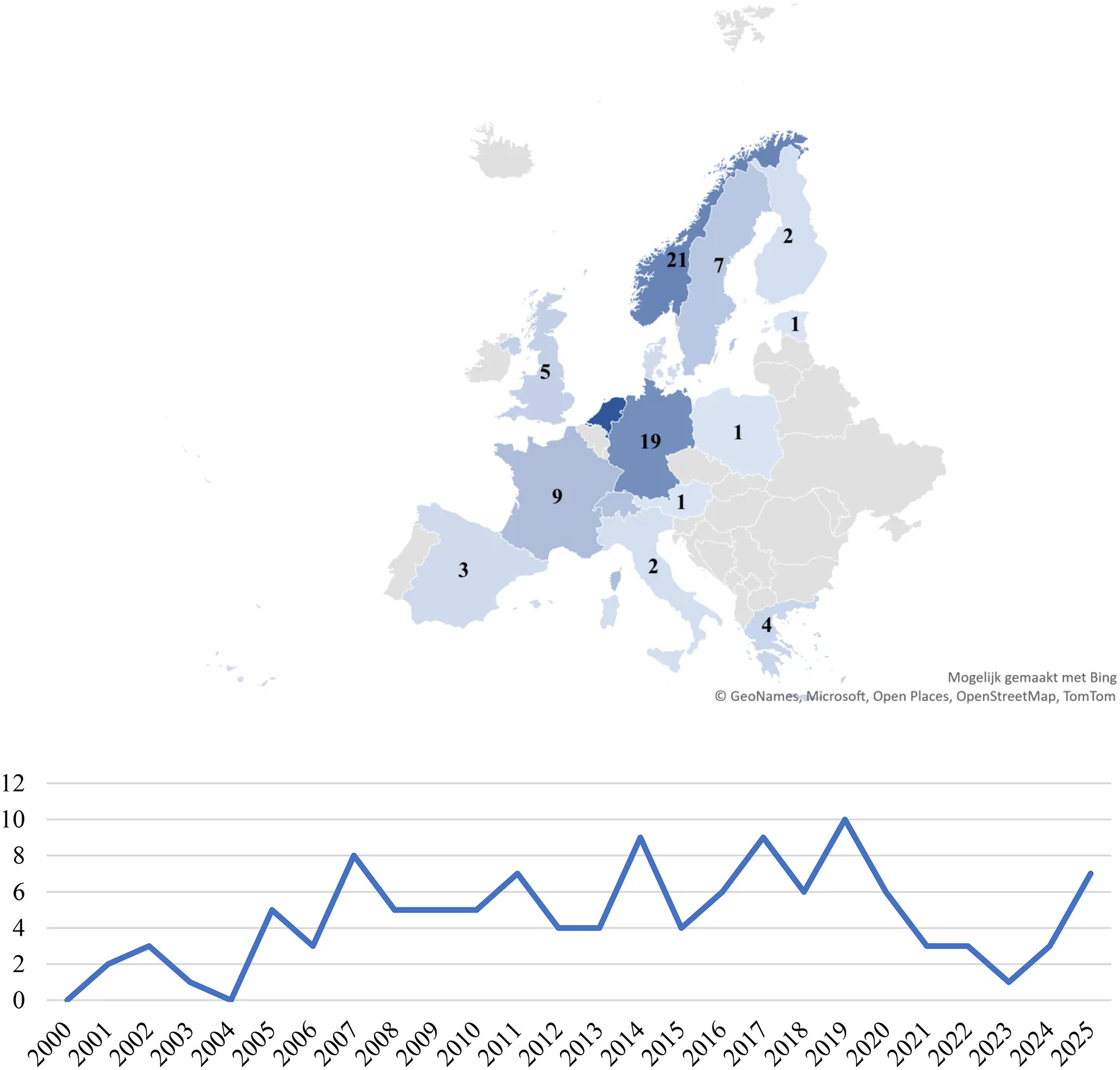
Geographic distribution on PROMs in severely injured trauma patient cohorts across Europe since 2000 including the distribution of publications per year

Baseline characteristics of included studies are shown in *Supplementary Table 1.* Most studies were conducted in the Netherlands (*n* = 31, 26.1%), followed by Norway (*n* = 21; 17.6%), and Germany (*n* = 19; 16.0%). A publication peak was seen between 2014 and 2019. The distribution of publications per country and the temporal trend in publication numbers is presented in Fig. 2. Most studies analysed general severely injured populations (*n* = 72; 60.5%). TBI cohorts were analysed in 20 studies (16.8%) and eighteen studies (15.1%) analysed cohorts of SCI patients. Smaller numbers of studies analysed patient cohorts with pelvic fractures (*n* = 3; 2.5%), general severely injured children (*n* = 2; 1.7%), TBI in children (*n* = 3; 2.5%), and severe burns (*n* = 1; 0.8%). Most studies were prospective cohort studies (*n* = 93; 78.2%). The median sample size was 123 (*IQR* 79–210) and median follow-up was 24 months (*IQR* 12–84).

### Quality assessment

Quality assessment results show that most studies demonstrated good quality in areas such as clear definition of inclusion criteria (*n* = 115; 96.6%), clear description of used PROMs (*n* = 115; 96.6%), data collecting routes (*n* = 98; 82.4%), and reporting complete outcome data (*n* = 94; 79%) (Table 1). Quality concerns were observed in reporting of data collecting personnel (*n* = 64; 53.8%), physical comorbidities (*n* = 43; 36.1%), potential recall bias (*n* = 40; 33.6%), and reporting psychiatric or mental comorbidities (*n* = 38; 31.9%).

**Table 1:** Summary of quality assessment across all 119 included studies

Quality assessment component	Low risk of bias - *n* (%)	High risk of bias - *n* (%)
Reporting incomplete outcome data	94 (79.0)	25 (21.0)
Inclusion criteria were clearly described	115 (96.6)	4 (3.4)
Data was part of a Trauma Registry	62 (52.1)	57 (47.9)
Data was part of a specific frequently published study cohort	98 (82.4)	21 (17.6)
Severely injured was clearly defined	115(96.6)	4 (3.4)
The response rate was clearly described	109 (91.6)	10 (8.4)
Potential recall bias was clearly described	40 (33.6)	79 (66.4)
Used PROMs were clearly described	115 (96.6)	4 (3.4)
Physical comorbidities were clearly described	43 (36.1)	76 (63.9)
Psychiatric comorbidities were clearly described	38 (31.9)	81 (68.1)
The route of data collection was clearly described	98 (82.4)	21 (17.6)
Data collecting personnel was clearly described	64 (53.8)	55 (46.2)
The responding person was clearly described	90 (75.6)	29 (24.4)

### Patient-reported outcome measures

**Fig. 3:**
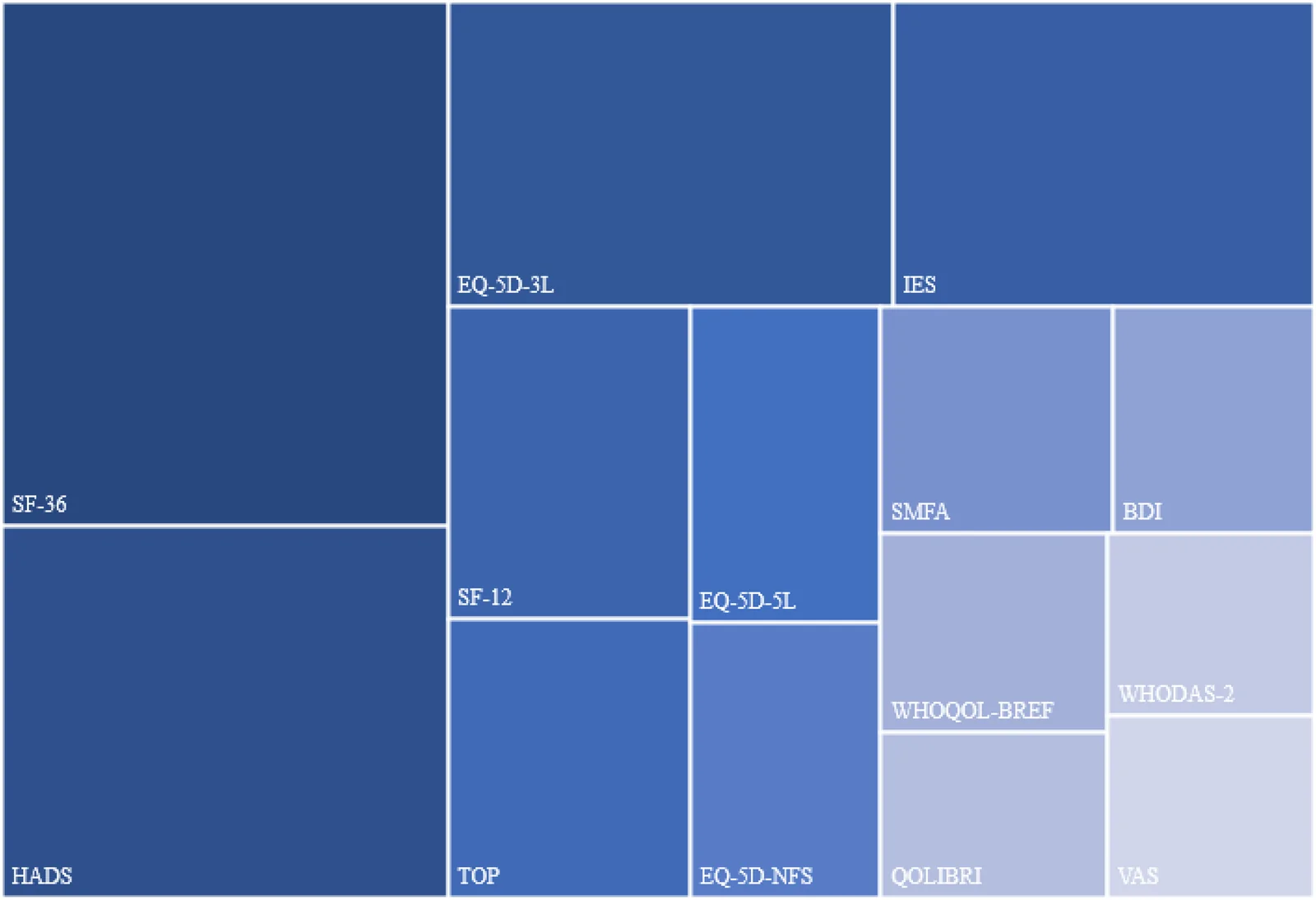
Tree map of the most used PROMs in European cohorts of severely injured patients. SF-36 = 36-Item Short Form Health Survey, SF-12 = 12-Item Short Form Health Survey, HADS = Hospital Anxiety and Depression Scale, EQ-5D-3 L and − 5 L = EuroQol-5d 3 L and 5 L, EQ-5D-NFS = EuroQol-5D-‘not further specified’, IES = Impact of Event Scale, TOP = Trauma Outcome Profile, SMFA = Short Musculoskeletal Function Assessment, BDI = Beck Depression Index, WHOQOL-BREF = World Health Organization Quality of Life Questionnaire, WHODAS-2 = World Health Organization Disability Assessment Schedule, QOLIBRI = Quality of Life after Brain Injury, VAS = Visual Analogue Scale

Overall, 94 different PROMs were used, which evaluated 277 follow-up timepoints. Most follow-up timepoints presented outcomes on HRQoL (*n* = 175, 63.2%). Psychological outcomes were presented in 64 follow-up timepoints (23.1%) and social participation in 38 follow-up timepoints (13.7%). A summary of the 15 most frequently used PROMs is shown in Fig. 3. Visualisation and frequencies of all 94 used PROMs are included in Supplemental Fig. 1.

### HRQoL

In total, 51 different PROMs were used to assess 175 HRQoL-related follow-up timepoints across 105 studies. These outcome data points were predominantly reported in general severely injured populations (*n* = 115; 65.7%), followed by cohorts of patients with TBI (*n* = 28; 16.0%) and SCI (*n* = 19; 10.9%). A limited number of follow-up timepoints were derived from cohorts of patients with pelvic fractures, severe burns, or paediatric populations. The most frequently used PROMs were the Short Form Health Survey (SF) instruments (SF-36 and SF-12 combined: *n* = 41; 23.4%) and EQ-5D (*n* = 33; 18.6%). A variety of other generic HRQoL questionnaires were used across the studies, with less frequent use and varying outcome definitions and follow-up timepoints.

Persistent impairments across multiple HRQoL domains were consistently reported after severe injury. Mobility limitations remained common throughout follow-up, with up to 56% of patients reporting mobility problems one year after trauma [8, 21–27] and 32% to 40% of patients continuing to report limitations five to six years later [11, 28, 29]. Up to ten years after trauma, physical functioning approached population norms in one study [30]. Similar recovery patterns were observed for self-care and usual activities. While both domains improved over time, persistent limitations remained common at long-term follow-up [8, 11, 22, 23, 27, 28]. Pain and discomfort also remained highly prevalent despite gradual improvement, with approximately 75% of patients reporting pain after one year and around 63% continuing to experience pain six years after trauma [11, 27].

Six months after trauma, EQ-5D index scores were reported in several studies and ranged between 0.67 and 0.71 [8, 23, 27, 31, 32]. At one year follow-up, a wider range of index scores was observed, varying from 0.57 to 0.80 [8, 22, 23, 26, 27, 31–38]. Beyond the first year, EQ-5D index scores generally plateaued, with scores reaching up to 0.87 at follow-up timepoints exceeding ten years after trauma [39, 40].

**Fig. 4:**
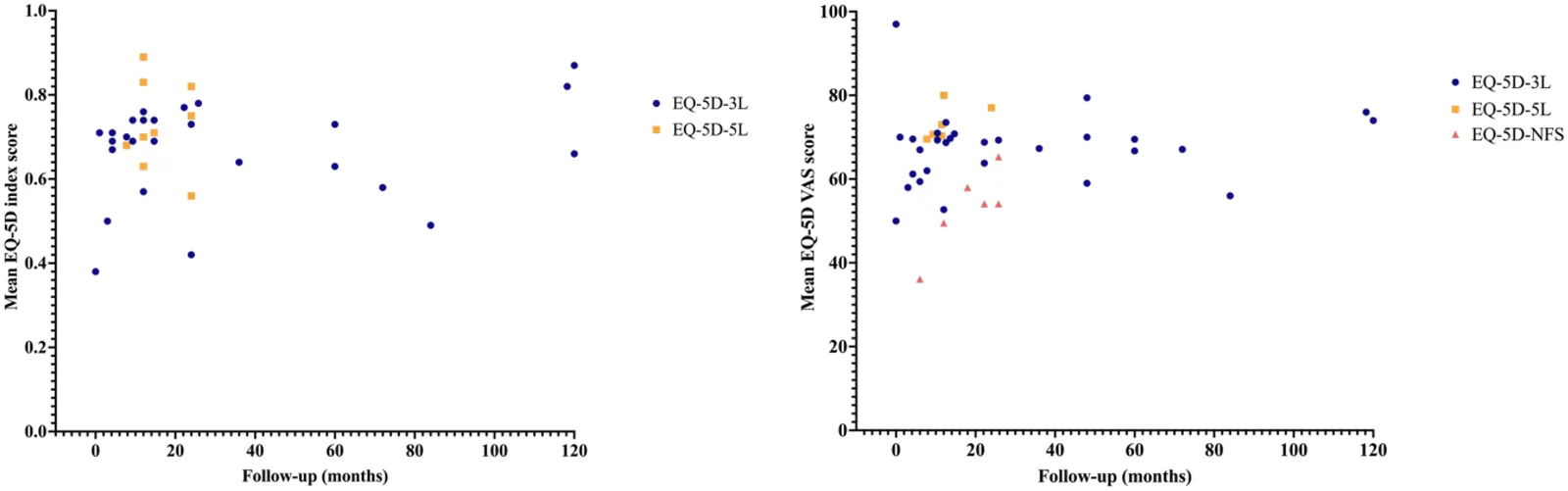
Trajectories of several PROMs outcomes over time. (**A**) EQ-5D index score and (**B**) EQ-5D VAS. *PROM* = Patient-reported Outcome Measure, *EQ-5D* = EuroQol-5D, *VAS* = Visual Analogue Scale

EQ-5D visual analogue scale (EQ-5D VAS) scores showed similar patterns over time. Six months after trauma, mean EQ-5D VAS scores ranged from 36 to 70 [8, 23, 27, 31, 32]. At one year after trauma, improvement in self-rated health was reported, with EQ-5D VAS scores reaching up to 80 in some cohorts [22]. Thereafter, no further improvement in EQ-5D VAS scores was observed at longer follow-up intervals. The trajectories of the EQ-5D index score and EQ-5D VAS over time are illustrated in Fig. 4A and B.

SF-36 physical health scores were consistently low, with reported mean values ranging from 16 to 53 in the early post-injury phase [12, 13, 41–44]. Over time, gradual improvement in physical health scores was observed across studies. At five to six years after trauma, physical and role-physical scores reached values up to 71, although substantial proportions of patients continued to report physical limitations [11, 28, 29].

**Fig. 5:**
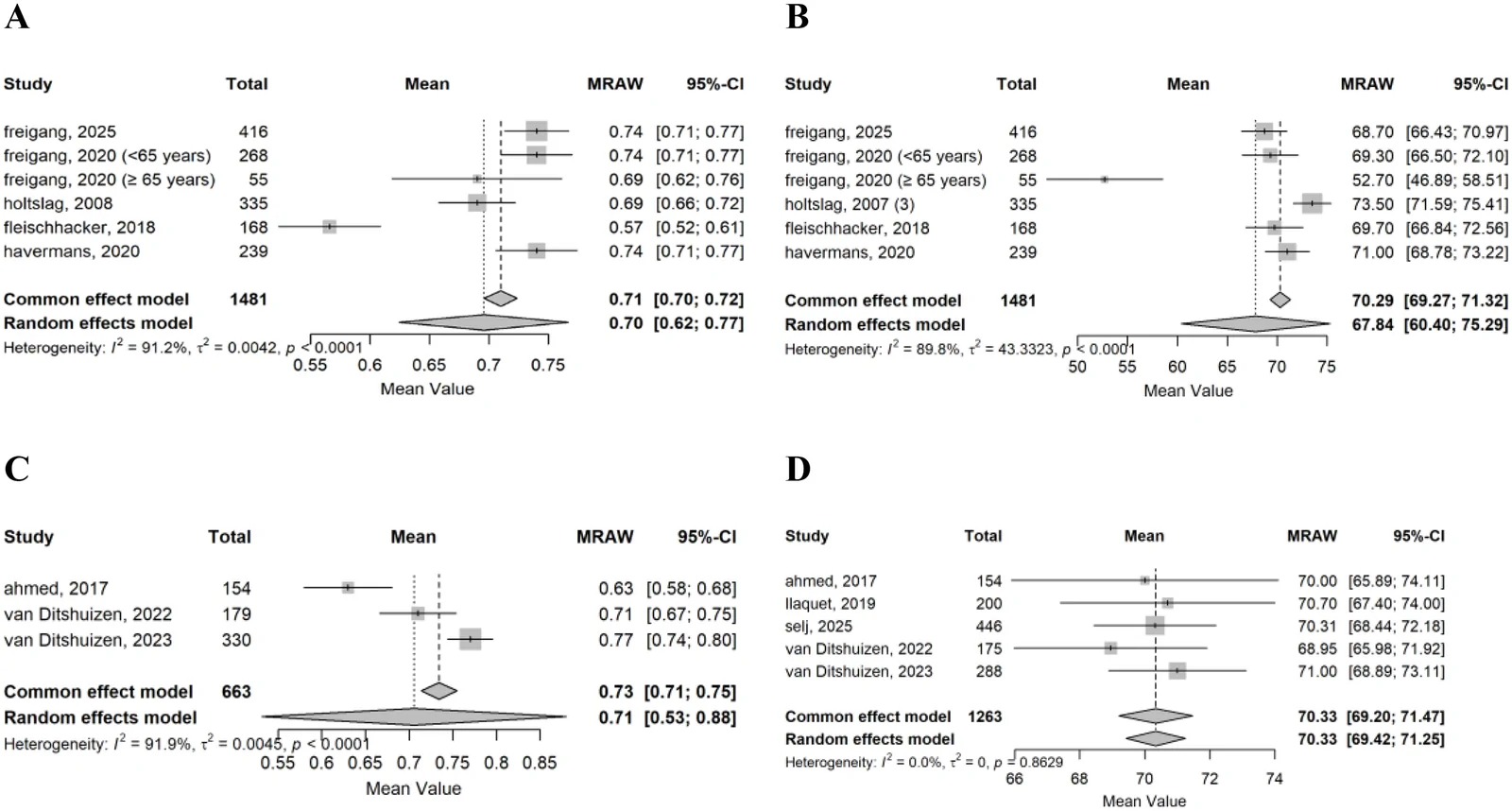
Forest plots of pooled outcome measures at 1 year follow-up, showing meta-analytic estimates for (**A**) EQ-5D-3 L index score, (**B**) EQ-5D-3 L VAS score, (**C**) EQ-5D-5 L index score, (**D**) EQ-5D-5 L VAS score. *EQ-5D* = EuroQol-5D, *VAS* = Visual Analogue Scale

As shown in Fig. 5A, the pooled mean EQ-5D-3 L index score at one year follow-up was 0.70 (*95% CI* 0.62–0.77). The pooled mean VAS score was 68 (*95% CI* 60–75), as illustrated in Fig. 5B. Substantial heterogeneity was observed at this follow-up timepoint (*I*^*2*^ = 90% for the index score and 91% for the VAS score). For the EQ-5D-5 L, the pooled mean index score at one year follow-up was 0.71 (*95% CI* 0.53–0.88), as shown in Fig. 5C, while the pooled mean VAS score was 70 (*95% CI* 69–71), as presented in Fig. 5D. Heterogeneity was high for the index score (*I*^*2*^ = 92%), whereas no heterogeneity was observed for the VAS score (*I*^*2*^ = 0%).

### Psychological outcomes

In total, 19 PROMs were used to assess psychological outcomes across 37 studies, comprising 64 follow-up timepoints. Psychological outcomes were most frequently reported in cohorts of general severely injured patients (*n* = 39; 60.9%), followed by cohorts of SCI patients (*n* = 14; 21.9%) and TBI patients (*n* = 11; 17.2%). The most used PROMs were the Hospital Anxiety and Depression Scale (HADS; *n* = 22; 34.4%) and the Impact of Event Scale (IES; *n* = 17; 26.6%). Other psychological questionnaires were used less frequently and showed substantial heterogeneity in outcome definitions and follow-up timepoints.

Symptoms of anxiety and depression were common after severe injury. At three months after trauma, clinically relevant anxiety symptoms were reported by 18% in general severely injured populations and by 21% of patients with TBI. Clinically relevant depression symptoms were reported by 9% and 15% of patients, respectively [45, 46]. At one year follow-up, anxiety symptoms persisted in 12% to 21% of patients, with higher proportions observed in TBI cohorts. Depressive symptoms were reported by 5% to 11% at this follow-up timepoint [45–48]. Long-term psychological impairments remained common with 48% up to 60% of patients reporting mental impairments ten years after trauma in two studies [9, 11, 49]. Symptoms of PTSD were frequently reported across trauma populations and showed a persistent course over time. One year after trauma, approximately 25% of patients in general severely injured populations met the diagnostic criteria for PTSD, while 21% reported subclinical PTSD symptoms [12, 50]. In TBI cohorts, PTSD symptoms were highly prevalent after discharge, with up to 88% of patients reporting trauma-related distress [51, 52].

**Fig. 6:**
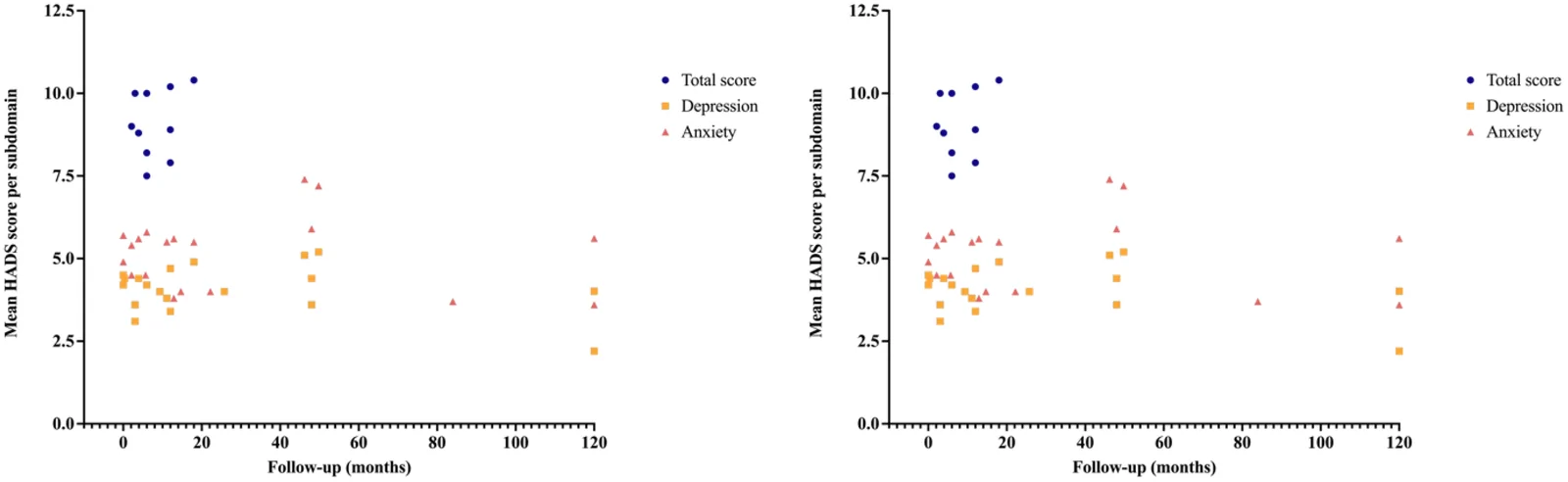
Trajectories of several PROMs outcomes over time. (**A**) HADS total score, depression, and anxiety and (**B**) IES total score, avoidance, intrusion, and hyperarousal. *PROM* = Patient-reported Outcome Measure, HADS = Hospital Anxiety Depression Scale, *IES* = Impact Event Scale

The trajectories of HADS total, anxiety, and depression scores over time are illustrated in Fig. 6A. Trajectories of IES total, avoidance, hyperarousal, and intrusion scores are shown in Fig. 6B.

**Fig. 7:**
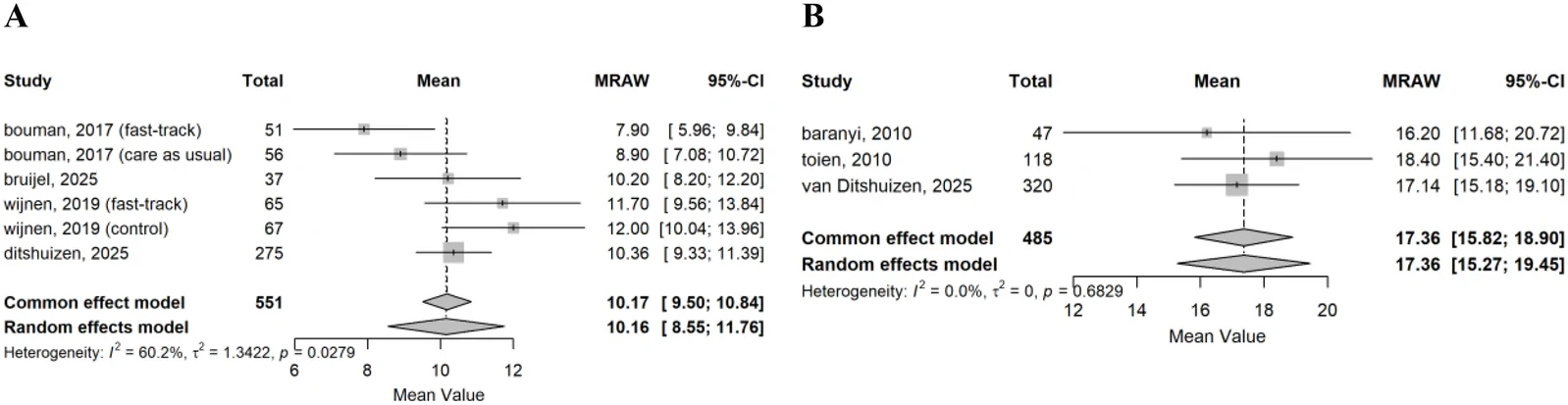
Forest plots of pooled outcome measures at 1 year follow-up, showing meta-analytic estimates for (**A**) HADS total score, and (**B**) IES total score. *HADS* = Hospital Anxiety Depression Scale, *IES* = Impact Event Scale

At one year follow-up, the pooled mean HADS total score was 10.2 (*95% CI* 8.6–11.8), with moderate heterogeneity (*I*^*2*^ = 60%) across studies, as shown in Fig. 7A. The pooled mean HADS depression subscale score was 6.3 (*95% CI* 3.6–8.9) and the pooled mean anxiety subscale score was 4.3 (*95% CI* 3.0-5.5). Heterogeneity was high for both subscales (*I*^*2*^ = 91% for depression and *I*^*2*^ = 80% for anxiety). For PTSD outcomes, the pooled mean IES total scores at one year follow-up was 17.4 (*95% CI* 15.3–19.5), with low heterogeneity (*I*^*2*^ = 0%), as illustrated in Fig. 7B.

### Social functioning and participation

In total, 29 PROMs were used to assess social participation across 27 studies, comprising 38 follow-up outcome data points. Social participation outcomes were most frequently reported in general severely injured populations (*n* = 15; 39.5%), followed by TBI (*n* = 13; 34.2%) and SCI cohorts (*n* = 9; 23.7%). One study assessed social participation in a paediatric severely injured patient cohort (2.6%). The most frequently used PROMs were Satisfaction with Life Scale (SWLS), Revised Life Orientation Test (LOT-R), and Brief Approach/Avoidance Coping Questionnaire (BACQ), all of which were used in three studies (7.9%). These PROMs were primarily used to assess subjective life satisfaction and coping-related aspects of social functioning.

Social functioning and participation were frequently affected after severe injury. Post-traumatic social growth, including improvements in interpersonal relationships, appreciation of life, and attitudes toward new possibilities, was reported [53]. In TBI cohorts, approximately 63% of patients reported satisfaction with life, and younger patients demonstrated better emotional and cognitive functioning compared to older patients [47, 54]. In SCI cohorts, social functioning was closely related to overall psychosocial functioning [55]. Among paediatric severely injured patients, behavioural and social problems were reported [56].

Meta-analysis was not feasible for social participation outcomes due to heterogeneity in measurement instruments and follow-up timepoints across the included studies.

## Discussion

Severely injured patients experience substantial and often persistent impairments across multiple outcome domains, including HRQoL, physical functioning, psychological health, life satisfaction, and social participation. Although gradual improvement over time is frequently observed, recovery often remains incomplete, with outcomes commonly below population norms [26, 37, 57–59]. These patterns are consistently reported across trauma subgroups and different trauma care settings, with considerable variation in magnitude and recovery trajectories.

Long-term reductions in HRQoL after severe injury have been described previously [60–62]. Evidence from previously published studies indicate that HRQoL after injury is not solely determined by injury characteristics but is strongly influenced by psychosocial factors such as mental health status, pain, coping strategies, self-efficacy, and perception of physical functioning [7, 49]. Persistent symptoms of depression, anxiety, and post-traumatic stress are common many years after injury and are strongly associated with reduced HRQoL [9, 49]. Previous studies suggest that psychological morbidity may be more common among patients with pre-existing mental health problems, persistent pain, reduced social support, impaired physical functioning, and females. Conversely, physical limitations are more strongly related to injury severity and musculoskeletal impairment, although considerable overlap exists between physical and psychological recovery [9, 12, 49, 54, 63]. In the current study, the EQ-5D was the most frequently used PROM across trauma subgroups and countries, with index and VAS scores generally remaining below previously reported population norms [64–74]. This underscores the lasting impact of severe injury on perceived health status. In addition, several included studies demonstrated that older patients generally reported poorer physical functioning, greater dependency in daily activities, and lower HRQoL than younger patients. This highlights age as an important factor influencing long-term recovery after severe injury [8, 74–76]. Importantly, the consequences of severe injury extend beyond patients themselves and affect significant others and caregivers, which highlights the broader societal burden of trauma.

Personality-related constructs were not included as an outcome domain in the present study, but they appear to be closely intertwined with HRQoL, psychological outcomes, and social participation. Personality traits such as neuroticism and extraversion have been shown to influence recovery trajectories and long-term HRQoL after traumatic injury [10, 77]. Higher neuroticism is consistently associated with poorer HRQoL, whereas extraversion and emotional stability appear to mitigate the negative impact of health conditions on mental health and overall HRQoL [10, 52, 55, 77]. These findings suggest that personality-related characteristics may partly explain heterogeneity in long-term PROMs and may act as mediators or moderators in the relationship between clinical covariates and patient-reported outcomes [78, 79].

A prominent methodological finding of the current study is the substantial heterogeneity in the use of PROMs across studies. Wide variation was observed in the selection of PROMs and in the reporting of outcomes, including differences in summary statistics and follow-up timepoints. These factors represent the most critical barrier to comparability and data synthesis. Consequently, the pooled estimates presented in this review provide an overall indication of patient-reported outcomes after severe injury. However, the substantial statistical heterogeneity observed across several meta-analyses limits their use as robust benchmark values for clinical practice or comparisons between trauma systems. In addition, heterogeneity in injury populations limited comparison between trauma subgroups, such as patients with TBI, SCI, musculoskeletal injuries, and general polytrauma. As injury patterns are likely to influence recovery trajectories and patient-reported outcomes, future research using harmonised PROM assessment should evaluate injury-specific outcomes in greater detail. Other process-related factors, such as integration with registry data and organisation of PROM administration, varied widely and are strongly dependent on local infrastructure. This limits their suitability for uniform standardisation. In contrast, greater consensus on which PROMs to use and how outcomes should be reported, appears both feasible and essential.

Recent international initiatives of the International Consortium for Health Outcomes Measurement Major Injury Working Group (ICHOM) have proposed standardised PROM sets for patients with major injury [80]. The findings summarised here support the relevance of such initiatives and emphasise the need for harmonised PROM selection and reporting practices to enable longitudinal comparisons, benchmarking between trauma systems, and data-driven quality monitoring. Adoption of such a core outcome framework could improve comparability between studies and trauma systems, reduce methodological heterogeneity, and facilitate pooled analyses of long-term recovery trajectories. In addition, consistent use of a core PROM set may support integration of patient-reported outcomes into trauma registries and routine clinical care pathways. Standardised PROM assessment could be incorporated into existing trauma registries by collecting PROMs at predefined follow-up intervals using digital questionnaires linked to existing registry infrastructure. This would facilitate longitudinal monitoring of recovery and identification of patients requiring additional rehabilitation or psychological support. This would strengthen the role of PROMs in monitoring recovery patterns and trauma care quality.

Several limitations should be acknowledged. Methodological quality varied across studies, with frequent risk of bias related to incomplete follow-up, heterogeneous populations, and selective outcome reporting. The inclusion of diverse trauma populations with different expected recovery trajectories further contributed to the observed heterogeneity and limited comparisons between injury subgroups. Also, the substantial heterogeneity between studies limits the interpretation and generalisability of the pooled meta-analytic estimates. In addition, severe injury was commonly defined using the ISS, despite its limited ability to capture long-term functional and patient-reported outcomes [81]. The predominance of observational designs, underrepresentation of Eastern European populations, and lack of individual patient data limited adjustment for confounding and restricted analyses of differences between patient groups and recovery patterns.

In summary, severe injury is associated with persistent impairments across multiple patient-reported outcome domains, which demonstrates that recovery extends far beyond physical healing. Psychological, social, and contextual factors play an important role in shaping long-term outcomes. Greater harmonisation of PROM selection and outcome reporting is essential to strengthen the evidence base and improve long-term trauma care.

## Conclusion

Severely injured patients experience substantial and often persistent limitations across multiple outcome domains, extending beyond physical recovery. Although patterns varied across trauma subgroups, recovery trajectories were generally characterised by incomplete restoration of pre-injury functioning, even at long-term follow-up. Too few results across European studies can be combined. There is a need for greater standardisation in outcome assessment because of heterogeneity in study designs, used PROM instruments, and follow-up timepoints. Furthermore, the lack of European-level integration for subgroup analyses and recovery trends limits the ability to draw comprehensive conclusions. A standardised routine integration of validated PROMs in clinical care pathways, with long-term follow-up, is crucial to help identify patients at risk for suboptimal recovery after severe injury. International initiatives such as the ICHOM set may provide an important framework for harmonised PROM selection and reporting, and their implementation in clinical practice and research is therefore encouraged.

## Supplementary Information

Below is the link to the electronic supplementary material.

Supplementary Material 1

## Data Availability

All data supporting the findings of this study are available within the paper and its Supplementary Information.
