# Supplementary material for: Patient-reported outcome measures within European cohorts of severely injured patients: a systematic review and meta-analysis
Source: Eur J Trauma Emerg Surg. 2026 Sep 4;52(1):285. doi: 10.1007/s00068-026-03325-y (PMC13545052; doi:10.1007/s00068-026-03325-y)
Supplement: Supplementary file 1 [file 68_2026_3325_MOESM1_ESM.docx]

**Supplements**

**Search Strategy**

| **Database search** | **Platform** | **Years of coverage** | **Records** | **Records after duplications removed** |
| --- | --- | --- | --- | --- |
| Medline ALL | Ovid | 1946 - present | 1,087 | 1,087 |
| Embase | Embase.com | 1971 - present | 1,699 | 1,005 |
| Web of Science Core Collection* | Web of Knowledge | 1975 - present | 737 | 186 |
| CINAHL Plus | EBSCO | 1982 - present | 585 | 186 |
| **Total** | | | **4,108** | **2,464** |

* Science Citation Index Expanded (1975-present); Social Sciences Citation Index (1975-present); Arts & Humanities Citation Index (1975-present) ; Conference Proceedings Citation Index- Science (1990-present); Conference Proceedings Citation Index- Social Science & Humanities (1990-present); Emerging Sources Citation Index (2005-present).

No other database limits were used than those specified in the search strategies.

Medline	1041

(Multiple Trauma/ OR Accidents, Traffic/ OR Wounds, Penetrating / OR Wounds, Nonpenetrating/ OR Air Ambulances / OR ((Accidents/ OR exp Emergency Medical Services/) AND "Wounds and Injuries"/) OR Brain Injuries, Traumatic/ OR Amputation, Traumatic/ OR Injury Severity Score/ OR (((multipl* OR severe* OR massive* OR major OR penetrat* OR blunt*) ADJ3 (trauma* OR injur* OR wound* OR burn*)) OR (trauma* ADJ3 amputat*) OR multitrauma* OR multiinjur* OR multi-trauma* OR multi-injur* OR polytrauma* OR polyinjur* OR poly-trauma* OR poly-injur* OR barytrauma* OR baryinjur* OR bary-trauma* OR bary-injur* OR ((traffic* OR car OR motorcycle* OR injur* OR emergen*) ADJ3 accident*) OR (helicopter* ADJ3 (trauma* OR emergenc*)) OR (brain ADJ3 trauma*) OR neurotrauma* OR neuro*-trauma*).ab,ti,kw. OR (traum* ADJ3 (injur* OR wound*)).ti.) AND (Patient Reported Outcome Measures/ OR Quality of Life/ OR * Return to Work/ OR * Anxiety/ OR * Fear/ OR * Stress Disorders, Post-Traumatic/ OR * Psychological Well-Being / OR * Mental Health/ OR ((Return to Work/ OR Anxiety/ OR Fear/ OR Stress Disorders, Post-Traumatic/ OR Psychological Well-Being / OR Mental Health/) AND ("Surveys and Questionnaires"/ OR Self Report/)) OR (((patient OR self) ADJ3 report* ADJ3 outcome*) OR prom OR proms OR (quality ADJ3 life) OR qaly* OR short-form OR shortform OR sf8 OR sf12 OR sf20 OR sf36 OR sf-8 OR sf-12 OR sf-20 OR sf-36 OR ((return-to-work* OR anxiety OR anxious* OR fear OR stress* OR ptsd OR wellbeing OR well-being OR mental-health OR psycholog*) ADJ10 (self-report* OR questionnaire* OR survey* OR assess*))).ab,ti,kw.) AND (exp Cohort Studies/ OR Controlled Before-After Studies/ OR Cross-Sectional Studies/ OR Registries/ OR (cohort* OR longitudinal* OR prospective* OR retrospective* OR cross-section* OR ((follow-up* OR outcome*) ADJ3 long*-term*) OR ((national* OR population* OR general*) ADJ3 survey*) OR (control* ADJ3 stud*)).ab,ti,kw. OR (follow-up* OR long*-term* OR register* OR registry* OR registries*).ti.) AND (exp Europe/ OR (Europe* OR Albania* OR Andorr* OR Armenia* OR Austria* OR Azerbaij* OR Balkan* OR Baltic-States* OR Estonia* OR Latvia* OR Lithuania* OR Belarus* OR Belgi* OR Benelux* OR Bosnia* OR Herzegovin* OR Bulgaria* OR Channel-Island* OR Croatia* OR Czech* OR England* OR France* OR French* OR German* OR Gibraltar* OR Britain* OR British* OR Greece* OR Greek* OR Hungar* OR Ireland* OR Irish* OR Isle-of-Man* OR Ital* OR Kosov* OR Liechtenstein* OR Luxembourg* OR Malta* OR Maltese* OR Moldova* OR Monac* OR Montenegr* OR Netherland* OR Dutch* OR Poland* OR Polis* OR Portug* OR Macedonia* OR Romania* OR Russia* OR San-Marin* OR Scandinavia* OR Denmark* OR Danish* OR Faroe* OR Finland* OR Finnish* OR Iceland* OR Norway* OR Norweg* OR Sweden* OR Swedish* OR Scotland* OR Scottish* OR Serbia* OR Slovakia* OR Slovenia* OR Spain* OR Spanish* OR Switzerland* OR Swiss* OR Ukraine* OR United-Kingdom* OR Vatican* OR Wales* OR Welsh* OR (Georgia* NOT (usa OR united-states))).ab,ti,kw.) NOT (news OR congres* OR abstract* OR book* OR chapter* OR dissertation abstract*).pt.

Embase	1658

('multiple trauma'/de OR 'traffic accident'/exp OR 'penetrating trauma'/de OR 'blunt trauma'/de OR 'helicopter emergency medical service'/de OR ((accident/de OR 'emergency health service'/de) AND injury/de) OR 'traumatic brain injury'/de OR 'traumatic amputation'/de OR 'injury severity'/mj OR 'injury scale'/mj OR (((multipl* OR severe* OR massive* OR major OR penetrat* OR blunt*) NEAR/3 (trauma* OR injur* OR wound* OR burn*)) OR (trauma* NEAR/3 amputat*) OR multitrauma* OR multiinjur* OR multi-trauma* OR multi-injur* OR polytrauma* OR polyinjur* OR poly-trauma* OR poly-injur* OR barytrauma* OR baryinjur* OR bary-trauma* OR bary-injur* OR ((traffic* OR car OR motorcycle* OR injur* OR emergen*) NEAR/3 accident*) OR (helicopter* NEAR/3 (trauma* OR emergenc*)) OR (brain NEAR/3 trauma*) OR neurotrauma* OR neuro*-trauma*):ab,ti,kw OR (traum* NEAR/3 (injur* OR wound*)):ti) AND ('patient-reported outcome'/de OR 'quality of life'/exp OR 'quality of life assessment'/exp OR 'mood disorder assessment'/exp OR 'anxiety assessment'/exp OR 'return to work'/mj OR anxiety/mj/exp OR fear/mj OR 'mental stress'/mj/exp OR 'posttraumatic stress disorder'/mj OR wellbeing/mj/exp OR 'mental health'/mj/exp OR (('return to work'/de OR anxiety/exp OR fear/de OR 'mental stress'/exp OR 'posttraumatic stress disorder'/de OR wellbeing/exp OR 'mental health'/exp OR 'psychological aspect'/de) AND ('health survey'/de OR questionnaire/exp OR 'self report'/de)) OR (((patient OR self) NEXT/3 report* NEAR/3 outcome*) OR prom OR proms OR (quality NEAR/3 life) OR qaly* OR short-form OR shortform OR sf8 OR sf12 OR sf20 OR sf36 OR sf-8 OR sf-12 OR sf-20 OR sf-36 OR ((return-to-work* OR anxiety OR anxious* OR fear OR stress* OR ptsd OR wellbeing OR well-being OR mental-health OR psycholog*) NEAR/10 (self-report* OR questionnaire* OR survey* OR assess*))):ab,ti,kw) AND ('cohort analysis'/de OR 'longitudinal study'/de OR 'prospective study'/de OR 'retrospective study'/de OR 'follow up'/de OR 'controlled study'/de OR 'cross-sectional study'/de OR register/mj OR (cohort* OR longitudinal* OR prospective* OR retrospective* OR cross-section* OR ((follow-up* OR outcome*) NEAR/3 long*-term*) OR ((national* OR population* OR general*) NEAR/3 survey*) OR (control* NEAR/3 stud*)):ab,ti,kw OR (follow-up* OR long*-term* OR register* OR registry* OR registries*):ti) AND (Europe/exp OR (Europe* OR Albania* OR Andorr* OR Armenia* OR Austria* OR Azerbaij* OR Balkan* OR Baltic-States* OR Estonia* OR Latvia* OR Lithuania* OR Belarus* OR Belgi* OR Benelux* OR Bosnia* OR Herzegovin* OR Bulgaria* OR Channel-Island* OR Croatia* OR Czech* OR England* OR France* OR French* OR German* OR Gibraltar* OR Britain* OR British* OR Greece* OR Greek* OR Hungar* OR Ireland* OR Irish* OR Isle-of-Man* OR Ital* OR Kosov* OR Liechtenstein* OR Luxembourg* OR Malta* OR Maltese* OR Moldova* OR Monac* OR Montenegr* OR Netherland* OR Dutch* OR Poland* OR Polis* OR Portug* OR Macedonia* OR Romania* OR Russia* OR San-Marin* OR Scandinavia* OR Denmark* OR Danish* OR Faroe* OR Finland* OR Finnish* OR Iceland* OR Norway* OR Norweg* OR Sweden* OR Swedish* OR Scotland* OR Scottish* OR Serbia* OR Slovakia* OR Slovenia* OR Spain* OR Spanish* OR Switzerland* OR Swiss* OR Ukraine* OR United-Kingdom* OR Vatican* OR Wales* OR Welsh* OR (Georgia* NOT (usa OR united-states))):ab,ti,kw) NOT ([conference abstract]/lim AND [2000-2022]/py)

Web of science	700

(TS=(((multipl* OR severe* OR massive* OR major OR penetrat* OR blunt*) NEAR/2 (trauma* OR injur* OR wound* OR burn*)) OR (trauma* NEAR/2 amputat*) OR multitrauma* OR multiinjur* OR multi-trauma* OR multi-injur* OR polytrauma* OR polyinjur* OR poly-trauma* OR poly-injur* OR barytrauma* OR baryinjur* OR bary-trauma* OR bary-injur* OR ((traffic* OR car OR motorcycle* OR injur* OR emergen*) NEAR/2 accident*) OR (helicopter* NEAR/2 (trauma* OR emergenc*)) OR (brain NEAR/2 trauma*) OR neurotrauma* OR neuro*-trauma*) OR TI=(traum* NEAR/2 (injur* OR wound*))) AND (TS=(((patient OR self) NEAR/2 report* NEAR/2 outcome*) OR prom OR proms OR (quality NEAR/2 life) OR qaly* OR short-form OR shortform OR sf8 OR sf12 OR sf20 OR sf36 OR sf-8 OR sf-12 OR sf-20 OR sf-36 OR ((return-to-work* OR anxiety OR anxious* OR fear OR stress* OR ptsd OR wellbeing OR well-being OR mental-health OR psycholog*) NEAR/10 (self-report* OR questionnaire* OR survey* OR assess*)))) AND (TS=(cohort* OR longitudinal* OR prospective* OR retrospective* OR cross-section* OR ((follow-up* OR outcome*) NEAR/2 long*-term*) OR ((national* OR population* OR general*) NEAR/2 survey*) OR (control* NEAR/2 stud*)) OR TI=(follow-up* OR long*-term* OR register* OR registry* OR registries*)) AND (TS=(Europe* OR Albania* OR Andorr* OR Armenia* OR Austria* OR Azerbaij* OR Balkan* OR Baltic-States* OR Estonia* OR Latvia* OR Lithuania* OR Belarus* OR Belgi* OR Benelux* OR Bosnia* OR Herzegovin* OR Bulgaria* OR Channel-Island* OR Croatia* OR Czech* OR England* OR France* OR French* OR German* OR Gibraltar* OR Britain* OR British* OR Greece* OR Greek* OR Hungar* OR Ireland* OR Irish* OR Isle-of-Man* OR Ital* OR Kosov* OR Liechtenstein* OR Luxembourg* OR Malta* OR Maltese* OR Moldova* OR Monac* OR Montenegr* OR Netherland* OR Dutch* OR Poland* OR Polis* OR Portug* OR Macedonia* OR Romania* OR Russia* OR San-Marin* OR Scandinavia* OR Denmark* OR Danish* OR Faroe* OR Finland* OR Finnish* OR Iceland* OR Norway* OR Norweg* OR Sweden* OR Swedish* OR Scotland* OR Scottish* OR Serbia* OR Slovakia* OR Slovenia* OR Spain* OR Spanish* OR Switzerland* OR Swiss* OR Ukraine* OR United-Kingdom* OR Vatican* OR Wales* OR Welsh* OR (Georgia* NOT (usa OR united-states)))) NOT DT=(Meeting Abstract OR Meeting Summary) AND LA=(English)

Cinahl	575

(MH Multiple Trauma OR MH Accidents, Traffic OR MH Wounds, Penetrating OR MH Wounds, Nonpenetrating OR ((MH Accidents OR MH Emergency Medical Services+) AND MH "Wounds and Injuries") OR MH Amputation, Traumatic OR MH Trauma Severity Indices OR T(((multipl* OR severe* OR massive* OR major OR penetrat* OR blunt*) N2 (trauma* OR injur* OR wound* OR burn*)) OR (trauma* N2 amputat*) OR multitrauma* OR multiinjur* OR multi-trauma* OR multi-injur* OR polytrauma* OR polyinjur* OR poly-trauma* OR poly-injur* OR barytrauma* OR baryinjur* OR bary-trauma* OR bary-injur* OR ((traffic* OR car OR motorcycle* OR injur* OR emergen*) N2 accident*) OR (helicopter* N2 (trauma* OR emergenc*)) OR (brain N2 trauma*) OR neurotrauma* OR neuro*-trauma*) OR (traum* N2 (injur* OR wound*)) OR AB(((multipl* OR severe* OR massive* OR major OR penetrat* OR blunt*) N2 (trauma* OR injur* OR wound* OR burn*)) OR (trauma* N2 amputat*) OR multitrauma* OR multiinjur* OR multi-trauma* OR multi-injur* OR polytrauma* OR polyinjur* OR poly-trauma* OR poly-injur* OR barytrauma* OR baryinjur* OR bary-trauma* OR bary-injur* OR ((traffic* OR car OR motorcycle* OR injur* OR emergen*) N2 accident*) OR (helicopter* N2 (trauma* OR emergenc*)) OR (brain N2 trauma*) OR neurotrauma* OR neuro*-trauma*) OR (traum* N2 (injur* OR wound*))) AND (MH Patient Reported Outcomes OR MH Quality of Life OR MM Job Re-Entry + OR MM Anxiety+ OR MM Fear+ OR MM Stress Disorders, Post-Traumatic+ OR MM Psychological Well-Being + OR MM Mental Health+ OR ((MH Job Re-Entry OR MH Anxiety OR MH Fear OR MH Stress Disorders, Post-Traumatic OR MH Psychological Well-Being OR MH Mental Health) AND (MH Questionnaires OR MH Self Report)) OR TI(((patient OR self) N2 report* N2 outcome*) OR prom OR proms OR (quality N2 life) OR qaly* OR short-form OR shortform OR sf8 OR sf12 OR sf20 OR sf36 OR sf-8 OR sf-12 OR sf-20 OR sf-36 OR ((return-to-work* OR anxiety OR anxious* OR fear OR stress* OR ptsd OR wellbeing OR well-being OR mental-health OR psycholog*) N10 (self-report* OR questionnaire* OR survey* OR assess*))) OR AB(((patient OR self) N2 report* N2 outcome*) OR prom OR proms OR (quality N2 life) OR qaly* OR short-form OR shortform OR sf8 OR sf12 OR sf20 OR sf36 OR sf-8 OR sf-12 OR sf-20 OR sf-36 OR ((return-to-work* OR anxiety OR anxious* OR fear OR stress* OR ptsd OR wellbeing OR well-being OR mental-health OR psycholog*) N10 (self-report* OR questionnaire* OR survey* OR assess*)))) AND (MH Prospective Studies + OR MH Controlled Before-After Studies OR MH Cross-Sectional Studies OR MH Registries, Trauma OR TI(cohort* OR longitudinal* OR prospective* OR retrospective* OR cross-section* OR ((follow-up* OR outcome*) N2 long*-term*) OR ((national* OR population* OR general*) N2 survey*) OR (control* N2 stud*)) OR (follow-up* OR long*-term* OR register* OR registry* OR registries*) OR AB(cohort* OR longitudinal* OR prospective* OR retrospective* OR cross-section* OR ((follow-up* OR outcome*) N2 long*-term*) OR ((national* OR population* OR general*) N2 survey*) OR (control* N2 stud*)) OR (follow-up* OR long*-term* OR register* OR registry* OR registries*)) AND (MH Europe+ OR TI(Europe* OR Albania* OR Andorr* OR Armenia* OR Austria* OR Azerbaij* OR Balkan* OR Baltic-States* OR Estonia* OR Latvia* OR Lithuania* OR Belarus* OR Belgi* OR Benelux* OR Bosnia* OR Herzegovin* OR Bulgaria* OR Channel-Island* OR Croatia* OR Czech* OR England* OR France* OR French* OR German* OR Gibraltar* OR Britain* OR British* OR Greece* OR Greek* OR Hungar* OR Ireland* OR Irish* OR Isle-of-Man* OR Ital* OR Kosov* OR Liechtenstein* OR Luxembourg* OR Malta* OR Maltese* OR Moldova* OR Monac* OR Montenegr* OR Netherland* OR Dutch* OR Poland* OR Polis* OR Portug* OR Macedonia* OR Romania* OR Russia* OR San-Marin* OR Scandinavia* OR Denmark* OR Danish* OR Faroe* OR Finland* OR Finnish* OR Iceland* OR Norway* OR Norweg* OR Sweden* OR Swedish* OR Scotland* OR Scottish* OR Serbia* OR Slovakia* OR Slovenia* OR Spain* OR Spanish* OR Switzerland* OR Swiss* OR Ukraine* OR United-Kingdom* OR Vatican* OR Wales* OR Welsh* OR (Georgia* NOT (usa OR united-states))) OR AB(Europe* OR Albania* OR Andorr* OR Armenia* OR Austria* OR Azerbaij* OR Balkan* OR Baltic-States* OR Estonia* OR Latvia* OR Lithuania* OR Belarus* OR Belgi* OR Benelux* OR Bosnia* OR Herzegovin* OR Bulgaria* OR Channel-Island* OR Croatia* OR Czech* OR England* OR France* OR French* OR German* OR Gibraltar* OR Britain* OR British* OR Greece* OR Greek* OR Hungar* OR Ireland* OR Irish* OR Isle-of-Man* OR Ital* OR Kosov* OR Liechtenstein* OR Luxembourg* OR Malta* OR Maltese* OR Moldova* OR Monac* OR Montenegr* OR Netherland* OR Dutch* OR Poland* OR Polis* OR Portug* OR Macedonia* OR Romania* OR Russia* OR San-Marin* OR Scandinavia* OR Denmark* OR Danish* OR Faroe* OR Finland* OR Finnish* OR Iceland* OR Norway* OR Norweg* OR Sweden* OR Swedish* OR Scotland* OR Scottish* OR Serbia* OR Slovakia* OR Slovenia* OR Spain* OR Spanish* OR Switzerland* OR Swiss* OR Ukraine* OR United-Kingdom* OR Vatican* OR Wales* OR Welsh* OR (Georgia* NOT (usa OR united-states)))) NOT PT(news OR congres* OR abstract* OR book* OR chapter* OR dissertation abstract

**Tables**

| **Table 1**: Overview of included studies reporting PROMs in European cohorts of severely injured patients since 2000. | | | | | | | | | | | | |
| --- | --- | --- | --- | --- | --- | --- | --- | --- | --- | --- | --- | --- |
| **Author**  **[ref]** | **Country** | **Year** | **Subgroup** | **Inclusion criteria** | **Exclusion criteria** | **Design** | **Sample size** | **Age,**  **mean (SD)/**  **median (IQR)** | **ISS,**  **Mean (SD)/**  **median (IQR)** | **PROMs** | **FU** | **Conclusion** |
| Vles  et al [1] | The Netherlands | 2005 | General | ISS ≥16 | Influent in Dutch | PCS | 166 | M: 33 (#)  F: 37 (#) | M: 23 (#)  F: 23 (#) | EQ-5D-NFS | 1y | -33% unable to return to work  -58% reporting pain/discomfort  -indicators: higher ISS, more affected body areas, females |
| Post  et al [2] | The Netherlands | 2006 | General | Age 18-65y, severely injured | Influent in Dutch | PCS | 53 | 37.3 (13.2) | 23.5 (21.0) | SIP-68, FAI | 2y | -87% returned to previous employment  -10% received disability benefits  -11% reporting severe disability |
| Holtslag  et al [3] | The Netherlands | 2006 | General | Age ≥16y, ISS ≥16, hospital trauma index ≥2 | Severe comorbidities | PCS | 186 | 37 (17) | 25 (11) | SF-36, GARS, SIP-136 | 1y | -permanent impairment occurred in 14%  - 28% had incomplete recovery  -50% reporting limitations in daily activities and mobility  -overall disability was mild-moderate |
| Holtslag  et al [4] | The Netherlands | 2007 | General | Age ≥16y, ISS ≥16 | - | PCS | 335 | 38 (17.1) | 25 (10.6) | SIP-136 | 1y | -overall disability was mild-moderate  -indicators: age and comorbidities |
| Holtslag  et al [5] | The Netherlands | 2007 | General | Age ≥16y, ISS ≥16 | - | PCS | 335 | 37.7 (17.1) | 24.9 (10.6) | EQ-5D-NFS | 1y | -48% reporting limitations in mobility  -55% reporting limitations in daily activities  -65% reporting limitations in cognitive complaints  -6% reporting pain  -indicators: SCI, TBI, extremity injuries, education level, and comorbidities |
| Holtslag  et al [6] | The Netherlands | 2007 | General | Age ≥16y, ISS ≥16 | - | PCS | 214 | 34.8 (11.6) | 25.0 (11.1) | GARS, HISC | 1y | -58.4% returned to full-time work  -21.5% returned to part-time work  -20.1% did not return to work  -indicators: SCI, hospital stay, discharge destination, and age |
| Holtslag  et al [7] | The Netherlands | 2008 | General | Age ≥16y, ISS ≥16 | - | PCS | 335 | 37.7 (17.1) | # | EQ-5D-NFS | 1y | -major trauma resulted in an average of 25 DALYs per patients  -RTA was the primary contributor |
| Ringburg et al [8] | The Netherlands | 2010 | General | Age >14y, ISS ≥16, multiple injuries, presentation at ER of level 1 trauma centre | Death on arrival | PCS | 246 | 40 (23-57) | 22 (17-29) | EQ-5D-NFS, HUI | 1y | -median EQ-5D index score was 0.73  -median EQ-5D VAS was 70  -18% reporting functional limitations  -60% reporting problems in ≥ EQ-5D domains  -indicators: females and comorbidities |
| Haagsma et al [9] | The Netherlands | 2012 | General | Age ≥16y, ISS ≥16, presentation at Level 1 trauma centre | Death on arrival | PCS | 226 | 42 (26-59) | 22 (17-29) | IES | 1y, 2y | -after 1y 23% reporting PTSD symptoms, of whom 79% reporting persistent PTSD symptoms  -after 2y 20% reporting PTSD symptoms  -indicators: females, comorbidities, head or extremity injury |
| Van Erp  et al [10] | The Netherlands | 2014 | General | ISS ≥16, treated at emergency care | Transferrals | PCS | 211 | 34.7 (11.6) | 25 (10.7) | SF-36, SIP-136, HLQ | 1y | -resuming paid work is not associated with reductions in unpaid activities |
| Van Delft-Scheurs  et al [11] | The Netherlands | 2014 | General | Age ≥18y, ISS ≥16, hospitalization between 2006 and 2008 | Cognitive impairments, influent in Dutch | CCS | 173 | 47 (19) | 21 (17-27) | HADS, WHOQOL-BREF, IES, CFQ | 3y | -psychological complaints are an important factor for decreased HRQoL |
| Van Delft-Scheurs  et al [12] | The Netherlands | 2014 | General | Age ≥18y, ISS ≥16 | Influent in Dutch | PCS | 173 | 47 (19) | 23 (8) | WHOQOL-BREF | 1y | -severely injured patients reported significantly worse HRQoL across all domains, except for social relations  -indicators: comorbidities, living alone, and not returning to work |
| Van Delft-Scheurs  et al [13] | The Netherlands | 2016 | General | Age ≥18y, ISS ≥16, fluent in Dutch | Cognitive impairments | CCS | 173 | 46 (19) | 21 (17-27) | HADS, WHOQOL-BREF, IES, SMFA, CFQ | 3y | -the SMFA correlated well with other measures of HRQoL and psychological well-being |
| Van Delft-Scheurs  et al [14] | The Netherlands | 2017 | General | Age ≥18y, ISS ≥16, fluent in Dutch | - | CCS | 173 | 46 (19) | 21 (17-27) | HADS, WHOQOL-BREF, SMFA | 1y, 2y, 3y, 4y | -limitations were mainly reported in lower extremity function resulting in decreased HRQoL  -mainly reported by patients with physical complaints |
| Ferree  et al [15] | The Netherlands | 2017 | General | Age ≥18y, ISS ≥16, hand injury | Deceased during or after admission, traumatic amputations, residency abroad | RCS | 52 | 44 (25-60) | 24 (20-34) | QDASH, PRQHE | 4y | -3.5% of patients had hand injury, mostly the metacarpal bones (48%) and carpal bones (33%)  -indicators: concomitant upper extremity, neurological injuries, and higher ISS scores had worse functional outcomes |
| Bouman  et al [16] | The Netherlands | 2017 | General | Polytrauma patients admitted to the AED, ISS ≥16, or complex multiple injuries, or complex pelvic ring injuries | Age <18y, influent in Dutch, severe substance abuse, severe comorbidities, no indication for clinical rehabilitation | PCS | 132 | FT: 44.7 (16.7)  CU: 42.0 (16.6) | FT: 22.1 (12.8)  CU: 29.4 (11.2) | SF-36, HADS | 3m, 6m, 9m, 1y | -both FT and CU rehabilitation programs showed significant improvements in functional status and HRQoL  -FT patients showed faster recovery after 6m, but no difference was found between both groups at 1y follow-up |
| Leijdesdorff  et al [17] | The Netherlands | 2018 | General | Age ≥18y, ISS ≥16, admission to Level 1 trauma centre in 2011-2012 | Deceased patients, self-limiting injuries, non-Dutch residents | PCS | 122 | 56.2 (21.1) | 22.2 (7.3) | SF-36, MFI-20, USER-P | 1-2y | -44% reported reduced physical health  -47% reported reduced mental health  -indicators: fatigue and societal participation, with severe TBI linked to worse mental health |
| Wijnen  et al [18] | The Netherlands | 2019 | General | Age ≥18y, severe injury (ISS ≥16, ≥2 injuries of which one life threatening, complex multiple injuries in both lower extremities, combination of lower and upper extremities) | Substance abuse, severe psychiatric comorbidities, influent in Dutch | RCT | 116 | FT: 44.7 (16.7)  CU: 42.0 (16.6) | FT: 22.1 (12.8)  CU: 29.4 (11.2) | SF-36, HADS | 3m, 6m, 9m, 1y | -FT rehabilitation improved functional outcomes but was more expensive than CU, with unfavourable cost-effectiveness ratios for QALYs |
| Havermans et al [19] | The Netherlands | 2020 | General | Age ≥18y, ISS ≥16, treatment in ICU, survival until discharge | Pathological fractures, influent in Dutch | PCS | 239 | 56.31 (19.3) | 20 (17-24) | EQ-5D-3L, HUI | 1w, 1m, 3m, 6m, 1y | -lower pre-injury health status, longer hospital stays, higher ISS, and comorbidities were prognostic factors for poorer HRQoL >1y post injury  -younger age and extremity injuries predicted short-term outcomes, unemployment and comorbidities mainly predicted long-term outcomes |
| Van der Vlegel  et al [20] | The Netherlands | 2021 | General | Age ≥18, ISS ≥16, admission to ward or ICU, survival up to discharge | Pathological fractures, absence of a permanent home address, influent in Dutch | PCS | 174 | 55 (40-66) | 20 (17-24) | EQ-5D-3L, iMCQ, iPCQ | 1w, 1m, 3m, 6m, 1y, 2y | -higher ISS and poorer health status were predictors of higher health care costs  -male sex was associated with increased productivity costs |
| Van Ditshuizen et al [21] | The Netherlands | 2022 | General | ISS ≥16, presentation at AED <48 hours after trauma | Death <1 year after trauma, transferrals, residency abroad | PCS | 182 | 52 (29-67) | 20 (17-25) | EQ-5D-5L | 1y | -most patients experience problems in all the EQ-5D-5L domains and cognition. Return to work status was associated with these outcomes |
| Van Ditshuizen et al [22] | The Netherlands | 2023 | General | Age ≥18y, ISS ≥16, hospitalized <48 post-injury, survival until discharge | Death on arrival, transfers | PCS | 335 | 55 (40-68) | 20 (17-25) | EQ-5D-5L | 1y, 2y | -no difference in HRQoL outcomes was reported between patients at Level 1 and Level 2/3 centres |
| Van Ditshuizen et al [23] | The Netherlands | 2025 | General | Age ≥18y, admission <48h after injury, survival to discharge | - | PCS | 335 | 55 (40-68) | 20 (17-65) | HADS, IES | 1y, 2y | -those treated at Level 1 trauma centres reported higher depression, anxiety, and PTSD rates |
| Schuckink Kool  et al [24] | The Netherlands | 2025 | General | Age ≥18y, lower extremity injury below mid-femur level | - | CCS | 87 | AMP: 29.8 (17.0)  REC: 45.2 (14.2) | # | LIMB-Q | 7y | -there was no outcome difference between amputation versus reconstruction  -indicators: age, FRI, and chronic pain |
| Van Baalen et al [25] | The Netherlands | 2007 | TBI | Age 18-85y, severe TBI, having a primary caregiver | Influent in Dutch or English, serious comorbidity | CCS | 51 | 34.2 (13.8) | # | SIP-68, FAI | 1y | -passive coping styles in primary caregivers of TBI patients is negatively associated with the patient's functional outcome  -patient's age and caregivers' coping style independently influenced restrictions in participation |
| Grauwmeijer et al [26] | The Netherlands | 2014 | TBI | Moderate to severe (GCS ≤12), non-penetrating trauma | Influent in Dutch, significant neurologic, oncologic, or other comorbidities | PCS | 97 | 32.8 (13) | # | SF-36 | 3m, 6m, 1y, 1.5y, 2y, 3y | -physical HRQoL improved over time  -while mental HRQoL remained stable  -indicators: mood and hospital stay |
| Bruijel  et al [27] | The Netherlands | 2025 | TBI | Age 21-80y, moderate to severe TBI (GCS ≤12, posttraumatic amnesia >24h, imaging abnormalities, loss of consciousness >30m), fluent in Dutch | Prior serious TBI other neurological conditions, sleep-awake disturbance, history of substance abuse, prior mental health disorders, pregnancy | PCS | 45 | 45.2 (16.7) | # | HADS, USER-P, FSS, PSQI | 3m, 6m, 1y, 1.5y | -after 1.5y high levels of fatigue and poor sleep quality were observed  -mood problems were associated with worse sleep quality and higher fatigue |
| Van Leeuwen  et al [28] | The Netherlands | 2012 | SCI | Age 18-65y, acquired SCI grade A-D, expected wheelchair dependency | SCI due to malignancy or progressive disease, psychiatric comorbidities, influent in Dutch | PCS | 61 | 45.3 (13.7) | # | SF-36, ICQ, SIP-68 | 5y | -activities and neuroticism were directly related to participation  -also appraisals and mental health predicted life satisfaction |
| Hermans  et al [29] | The Netherlands | 2019 | Pelvic injury | Age 18-80y, pelvic ring fractures due to HET | Osteoporotic fractures, LET, death on arrival, influent in Dutch | PCS | 136 | 39 (17) | 22.8 (14) | EQ-5D-NFS, SMFA | 8y | -the mean MPS was 74  -the EQ-5D index was 0.81 for tile A, 0.87 for tile B, and 0.82 for tile C  -the mean SMFA index was 24 |
| Nooijen  et al [30] | The Netherlands | 2017 | SCI | Age 18-65y, subacute SCI, wheelchair dependent, able to handcycle | Influent in Dutch, progressive disease, psychiatric comorbidity | RCT | 20 | 44 (15) | # | SF-36 | BL, discharge, 6m, 1y | -behavioural interventions promoting physical activity improved social participation and significantly reduced cardiovascular risk factors |
| Janssens  et al [31] | The Netherlands | 2009 | Children (general) | Age <16y, ISS ≥16 | - | PCS | 28 | 8.2 (4.7) | 24.7 (11.7) | EQ-5D-NFS, PedsQL | 6-8y | -lowest HRQoL scores were seen in adolescents with SCI or TBI |
| Janssens  et al [32] | The Netherlands | 2009 | Children (general) | Age <16y, ISS ≥16 | - | PCS | 28 | 8.2 (4.7) | 24.7 (11.7) | SF-12, SDQ | 6-8y | -80% showed good recovery  -57% having no impairments  -25% showed behavioural problems |
| Snekkevik  et al [33] | Norway | 2002 | General | Age 18-68y, severe trauma (multiple traumata in 1 body region or ≥1 body region affected) | Neuropsychological deficits due to TBI | PCS | 26 | 31 (18-68) | # | HADS, VAS, SOC-13, GHQ-20 | 1-3y | -the median SOC remained stable  -SOC did not predict long-term life satisfaction or psychological well-being  -SOC was closely associated with overall life satisfaction and psychological distress |
| Soberg  et al [34] | Norway | 2006 | General | Age 18-67y, NISS ≥16, ≥2 injuries classified according to the AIS system | Former multiple injuries, burn injuries, substance addiction, severe psychological disease, aphasia, influent in Norwegian | PCS | 101 | 35.3 (14) | 27.5 (11.4) | SF3-36, WHODAS-2, COG | BL, 1y, 2y | -43% achieved full return to work (average time 12.8m)  -indicators: lower education, longer hospital/rehabilitation stay, and low social functioning shortly after returning home |
| Soberg  et al [35] | Norway | 2007 | General | Age 18-67y, NISS ≥16, ≥2 injuries classified according to the AIS system | Former multiple injuries, burn injuries, substance addiction, severe psychological disease, aphasia, influent in Norwegian | PCS | 97 | 34.5 (13.5) | 28.1 (11.3) | SF-36, WHODAS-2, COG, MHLC, BACQ | BL, 1y, 2y | -there were long-lasting functional impairments across multiple life domains  - physical and social functioning were mostly affected |
| Ulvik  et al [36] | Norway | 2007 | General | Age >18y | Foreigners | PCS | 210 | 39 (17) | 25 (4-54) | EQ-5D-NFS | 2-7y | -74% reported HRQoL impairments, of whom 16% experienced severe problems |
| Quale  et al [37] | Norway | 2009 | General | Age 16-68y | Severe cognitive or psychiatric disorders, influent in Norwegian | PCS | 79 | 37 (14.3) | 25.6 (12.3) | HADS, IES, AEE | 2y | -6% met the diagnostic criteria for PTSD  -9% had subsyndromal PTSD  -no correlation was found between ISS/trauma mechanism on PTSD symptoms. |
| Quale  et al [38] | Norway | 2010 | General | Age 16-68y, admitted to SRH | Severe cognitive, psychiatric or medical deficits, influent in Norwegian | PCS | 80 | 39.1 (15.6) | 31.9 (13.5) | HADS, IES, LOT-R | 1y | -resilience was the most common adaption pattern to severe injury  -trait effect played a key role in determining psychological outcome  -indicators: trait negative and positive effect |
| Soberg  et al [39] | Norway | 2010 | General | Age 18-67y, NISS ≥16, ≥2 injuries classified in the AIS scoring system, fluent in Norwegian | Former major trauma, substance addiction, severe psychologic disease | PCS | 99 | 35.3 (14.2) | 34.9 (12.7) | SF-36, WHODAS-2, BACQ, PTSS-10 | BL,1y, 2y | -20% had PTSD symptoms  -27% had depression symptoms  -indicators: females, younger age, avoidant coping, pain, mental health, and cognitive functioning |
| Tøien  et al [40] | Norway | 2010 | General | Age 18-75y, admitted to the hospital after trauma, fluent in Norwegian | Patients living abroad, self-inflicted injuries, severe cognitive or psychiatric disorders, untraceable home address | PCS | 118 | 40 (#) | 23.1 (#) | HADS, IES, VAS | BL, 3m, 1y | -patients experienced psychological distress, including posttraumatic stress symptoms, anxiety, and depression |
| Tøien  et al [41] | Norway | 2011 | General | Age 18-75 y, admitted to the hospital after trauma, fluent in Norwegian | Patients living abroad, self-inflicted injuries, severe cognitive or psychiatric disorders, untraceable home address | PCS | 103 | 42.3 (#) | 10 (#) | SF-36, HADS, IES, LOT-R | 3m, 1y | -HRQoL improved significantly but remained below population norm |
| Vassend  et al [42] | Norway | 2011 | General | NISS ≥16, ≥2 injuries classified in the AIS scale, SCI, fluent in Norwegian | Severe cognitive, psychiatric, or medical complaints | PCS | 75 | 45 (14.5) | 33 (14.1) | NRS, HADS, IES, SIP-136, LOT-R | BL, discharge, 4y | -dispositional optimism and pain were important predictors of health status |
| Soberg  et al [43] | Norway | 2012 | General | Age 18-67y, NISS ≥16, ≥2 injuries classified in the AIS scoring system, fluent in Norwegian | Previous multiple traumata, substance addiction, severe psychological disease, aphasia | PCS | 78 | 35.3 (14.0) | 34.6 (12.6) | SF-36, WHODAS-2, BACQ | BL, 1y, 2y, 5y | -physical and mental health improved over time but remained below population norms |
| Soberg  et al [44] | Norway | 2015 | General | Age 18-67y, NISS ≥16, ≥2 injuries classified according to the AIS scoring system, hospital admission <48h after injury | History of multiple injuries, burns, substance abuse, severe psychological disorders, progressive malignancy | PCS | 104 | 43 (74.1) | 33.7 (13) | SF-36, COQ, BACQ | 1y, 2y, 5y, 10y | -long term after injury physical health was below population norms, mental health was similar to population norms |
| Selj  et al [45] | Norway | 2025 | General | Age ≥18y, NISS ≥9, admission within 72h, hospitalized for ≥2d | Non-Norwegian residents, influent in Norwegian or English | PCS | 446 | 51.7 (17.8) | 24.2 (12.0) | EQ-5D-5L | 6m, 1y | -HRQoL remained stable, with improvements in usual activities and anxiety/depression  -unmet healthcare and social support needs were associated with worse HRQoL |
| Ringdal  et al [46] | Norway | 2025 | General | NISS ≥12, single head injury with AIS ≥3 | Chronic subdural hematoma, drowning accidents, inhalation injury, asphyxiation | PCS | 2614 | 41 (21-62) | 5 (1-10) | EQ-5D-5L | 6m, 1y | -47.2% reported anxiety and depression  -26.4% did not return to work |
| Andelic  et al [47] | Norway | 2009 | TBI | Age 16-55y, moderate (GCS 9-12) to severe (GCS ≤8) TBI at time of admission | Previous TBI, neurological or psychiatric disorders, severe substance abuse, associated SCI | RCS | 62 | 31.2 (10.9) | # | SF-36, BDI | >10y | -48% showed good recovery  -44% had moderate disability  -58% were unemployed  -HRQoL was below population norms |
| Soberg  et al [48] | Norway | 2013 | TBI | Age ≥16y, admission <72h post-injury, severe TBI (GCS ≤8) | Chronic subdural hematoma, severe physical or psychological comorbidities, substance abuse | PCS | 126 | 38.9 (17.8) | 27.2 (11.8) | RPQ, HADS, QOLIBRI | 3m, 1y | -the mean HRQoL by using the QOLIBRI was 68.5  -key predictors: employment status, global functioning, post-concussion symptoms, and psychological distress. |
| Anke  et al [49] | Norway | 2015 | TBI | Severe TBI (GCS ≤8), admission to 1 of 4 trauma centres <24h after trauma | Psychiatric or neurological comorbidities, severe substance abuse, patients living abroad | PCS | 163 | 40.1 (18.6) | 28.1 (11.7) | RPQ, SWLS, HADS | 3m, 1y | -60% reported functional improvements  -85% were independent in daily activities  -63% were satisfied with life |
| Løke  et al [50] | Norway | 2022 | TBI | TBI, admission to the neurosurgery department at OUH between 2018 and 2020 | Severe neurological or mental disorders, substance abuse, influent in Norwegian or English | PCS | 96 | 45 (13.9) | # | NRS, RPQ, NEO-FFI, LOT-R, FSS, CFS, HSC, BIS/BAS, UCLA Loneliness Scale, RSA, GSCL | 6m | -physical robustness, somatic vulnerability, and ISS were significantly associated with fatigue. |
| Lidal  et al [51] | Norway | 2008 | SCI | tSCI, admission at SRH between 1961 and 1982 | - | CSS | 165 | 23 (9.7) | # | SF-36 | >10y | -HRQoL was significantly lower in the domains physical functioning, bodily pain, general health, and vitality |
| Lidal  et al [52] | Norway | 2013 | SCI | tSCI, admission at SRH between 1961 and 1982 | - | CSS | 153 | 23.3 (9.6) | # | HADS, FQ | >10y | -25% reported fatigue  -higher fatigue scores were associated with depression |
| Jakimovska et al [53] | Norway | 2017 | SCI | Traumatic SCI, admission at SRH between 1961 and 1982 | - | RCS | 147 | 23 (10) | # | CHQ-20 | >10y | -34% were satisfied with SCI-specific follow-up services  -23% experienced psychological distress |
| Pirente  et al [54] | Germany | 2001 | General | Age ≥16y, severely injured, treated in Köln between 1993-1994 | Another traumatic injury event, severe psychiatric comorbidity | PCS | 43 | 38 (19-61) | 21 (5-45) | SF-36, TOP, BDI | 1y | -48.8% reported moderate-severe -25% reported anxiety or depression symptoms |
| Stalp  et al [55] | Germany | 2002 | General | ISS ≥16, ≥2y after blunt injury, treated in a Level 1 trauma centre | - | PCS | 196 | 36 (13) | 24 (6) | SF-12, SMFA, HASPOC | 2y | -patients with blunt multiple injuries generally had fair or good outcomes  -limitations were primarily reported in extremities, mainly below the knee  -41% with lower extremity injury reported moderate-severe restrictions |
| Janssen  et al [56] | Germany | 2008 | General | Age 18-75y, seriously injured (AIS of the 2 worst injuries ≥6), mentally orientated | Severe cranial injury (AIS >3 and coma >24h), suicide attempt, victims of violent crimes, previous mental disorders, influent in German | RCS | 90 | 42.3 (12.9) | 22.9 (9.5) | SF-36, TOP, POLO-chart | 4y | - SF-36 scores were significantly lower than the German norm  -indicators: higher age, lower socioeconomic status, living alone, ISS, and extremity injury were predictors |
| Lefering  et al [57] | Germany | 2011 | General | Age 18-65y, >6m since injury, fluent in German | Accident occurred <1999 | PCS | 172 | 38.8 (14.4) | 26.7 (11.2) | IES, TOP, STAI, F-SOZU-K-22 | 2y | -TOP demonstrated good internal consistency and construct validity, with strong correlations to external measures such as the BDI and SF-36 |
| Simmel  et al [58] | Germany | 2013 | General | Age 16-60y, ISS ≥25, treatment at BG Trauma Center Murnau between 2000-2005 | SCI, severe neurological or psychiatric comorbidities | PCS | 127 | 36 (#) | 35.6 (26-75) | EQ-5D-NFS, SF-36, TOP | 5y | -HRQoL remained reduced compared to the general population  -indicators: pre-traumatic factors  and posttraumatic factors were identified as negatively impacting long-term HRQoL |
| Kaske  et al [59] | Germany | 2014 | General | Age ≥18y, treatment at Cologne Merheim Medical Center between 2008-2019, registered in the TR-DGU | Serious cognitive comorbidities, vegetative state, death | PCS | 129 | 43.9 (17.5) | 21.5 (13.3) | EQ-5D-NFS, SF-36, TOP, POLO-chart | 2y | -62% reported persistent pain  -64% had severe functional deficits |
| Kaske  et al [60] | Germany | 2016 | General | Age ≥18y | Cognitive impairments | PCS | 210 | 43.6 (18.1) | 19.1 (12.9) | TOP | 2y | -59% reported severe persisting pain  -53% receiving pharmacological pain management |
| Zwingmann et al [61] | Germany | 2016 | General | Polytrauma patients | - | PCS | 147 | 40 (19) | 28 (11) | EQ-5D-NFS, SF-36, TOP, BDI | 6y | -patients reported persistent pain, functional disabilities, and psychological after-effects |
| Falkenberg et al [62] | Germany | 2017 | General | Age 3-60y, blunt injury of polytrauma | Age ≤10y at time of injury | RCS | 149 | 30.0 (12.2) | 21.1 (9.3) | HADS, IES, SF-12, HASPOC, PDS, SSES-9 | >10y | -45.9% reported symptoms of mental impairments |
| Fleischhacker  et al [63] | Germany | 2018 | General | Age ≥18y, treatment in ICU, survival >500d | Influent in German, treatment in closed psychiatric ward, severe neurological or psychiatric disease | PCS | 168 | 42.6 (19.6) | 23.6 (#) | EQ-5D-3L | 12m, 3y, 5y, 6y, 7y | -43.5% had a poor EQ-5D index (< 0.6)  -28% had a low EQ-5D VAS (≤ 50) |
| Angerpointner  et al [64] | Germany | 2019 | General | All patients treated in the trauma network of eastern Bavaria | Age <18y, patients treated in a Level 3 centre | PCS | 501 | 45.5 (22.2) | 16 (13) | EQ-5D-NFS | 6m, 1y, 2y | -the EQ-5D index improved from 0.71 (*SD* 0.31) at 6m to 0.76 (*SD* 0.27) at 24m (*p* < 0.001), but remained below the reference value (0.9) |
| Freigang  et al [65] | Germany | 2020 | General | Age ≥16y, ISS ≥16 | - | PCS | 405 | <65y: 43.0 (24.3-53.0)  >65y: 73.0 (70.0-78.0) | <65y: 22 (18-30)  >65y: 20 (17-27.5) | EQ-5D-3L | 6m, 1y, 2y | -patients ≥65y reported more difficulties in all EQ-5D domains |
| Kalbas  et al [66] | Germany | 2022 | General | Age 30-60y | Amputations, paraplegia | PCS | 337 | 25.4 (11.7) | 20.3 (9.3) | PTGI | >10y | -96.5% reported post traumatic growth  -significant improvements were seen in in relationships, life appreciation, and attitudes towards new possibilities |
| Jaekel  et al [67] | Germany | 2024 | General | Age 18-75y, ISS ≥16, treatment in one of 6 participating GDU hospitals | Transfer <48h | PCS | 594 | # | # | SF-12 | 1y | -physical QoL was significantly better in younger patients and worsened with higher ISS  -mental QoL showed no difference by injury severity  -82.1% were satisfied with treatment outcomes  -45.2% returned to full-time work |
| Freigang  et al [68] | Germany | 2025 | General | Age ≥16y, ISS ≥16 | Secondary admission | PCS | 416 | # | 22 (17-29) | EQ-5D-5L | 6m, 1y, 2y | -female patients reported more anxiety and depression  -females ≤39y showed greatest improvement in HRQoL |
| Schönenberg et al [69] | Germany | 2014 | SCI | Age 18-60y, thoracic or lumbosacral traumatic SCI, fluent in German | Previous or current psychosis, TBI, SCI due to suicide attempt | RCS | 102 | 37.83 (12.37) | # | IES, PTCI, ADS-K, TLEQ, brief COPE | 4y | -46.1% reported clinically relevant depressive symptoms  -88% reported PTSD symptoms  -indicators: negative trauma-related self-appraisals and time since injury |
| Van Loon  et al [70] | Germany | 2011 | Pelvic ring | Open book pelvic injury (B1), admission to a Level 1 trauma centre between 1996-2008 | - | PCS | 32 | 44 (33-60) | 21 (16-32) | SF-36 | >10y | -the mean Majeed score was 95.7  -SF-36 scores on the domains role physical and physical function were lower compared to the general population |
| Gojowy  et al [71] | Germany | 2019 | Burns | Age ≥16y, TBSA ≥20%, ≥10y follow-up | Death during or after hospital stay, non-German citizen | PCS | 42 | 37 (17) | # | SF-36, BSHS-B | >10y | -the HRQoL was like the general population |
| Riuttanen  et al [72] | Finland | 2024 | General | Age ≥18y, NISS ≥16, alive 1y post-injury, valid Finnish IDN | - | PCS | 325 | 53.6 (17.7) | 17 (11) | EQ-5D-3L | 1y | -ISS had no significant association with decreased HRQoL  -indicators: SCI, lower lever of education, length of ICU stay |
| Kyösti  et al [73] | Finland | 2024 | TBI in children | Age <18y, treatment in ICU for TBI | - | RCS | 150 | 12.0 (4.8) | # | RAND-36 | >10y | -49% reported poor HRQoL |
| Vardon-Bounes  et al [74] | France | 2021 | General | Age >16y, ISS ≥9, treatment in ICU | Survival <30d, minors | PCS | 55 | 46 (17.76) | 18 (9-41) | SF-36, NHP | 5y | - 26% was unable to return to work  -36.4% had a disability status |
| Quintard  et al [75] | France | 2002 | TBI | Severe TBI (GCS ≤8), alive and tractable | Patients or families who refused to participate | PCS | 79 | 40.5 (17) | # | SWLS | 7y | -65-85% was independent in daily functioning  -35-55% was independent in social life  -67% reported satisfaction in autonomy  -41% reported satisafaction in financial status |
| Mailhan  et al [76] | France | 2005 | TBI | Age 16-65y, severe TBI (GCS ≤8) | Substance abuse, persistent vegetative state, severe communication impairments | CCS | 75 | 29.68 (10.20) | # | SQLP, NRS-R | 2y | -life satisfaction was generally low  -dissatisfaction in cognitive functions, physical abilities, and self-esteem was reported |
| Jourdan  et al [77] | France | 2013 | TBI | Age ≥15y, severe TBI (GCS ≤8) | - | PCS | 134 | 36.0 (16.3) | # | DEX | 1y | -19% had good recovery  -43% had moderate recovery  -38% had persistent severe disability  -42% returned to work with 28% did not change jobs |
| Jourdan  et al [78] | France | 2016 | TBI | Age ≥15y, severe TBI (GCS ≤8 before hospital admission) | Other causes for coma | PCS | 147 | 32.5 (14.2) | # | HADS, QOLIBRI, NRS-R | 4y | -32% reported severe disability  -40% reported moderate disability  -28% reported good recovery  -common complaints were fatigue, headache, cognitive complaints, anxiety (43%), and depression (25%) |
| Azouvi  et al [79] | France | 2016 | TBI | Age ≥15y, severe TBI (GCS ≤8) | Other causes for coma | PCS | 85 | 31.7 (12.9) | # | QOLIBRI | 4y | - disability was influenced by mood, cognition, ISS, and physical impairments  -HRQoL was influenced by mood and cognition, with age and education level indirectly affecting both outcomes |
| Chesnel  et al [80] | France | 2018 | TBI | Age ≥16y, severe TBI (GCS ≤8) | - | PCS | 90 | 32.5 (1.4) | # | HADS, DEX, QOLIBRI, NRS-R, BICQ | 4y | -lack of awareness of difficulties was associated with the burden perceived by their relatives (*R* = 0.5; *p* < 0.001). |
| Bouzat  et al [81] | France | 2019 | TBI | Adult patients, treatment in ICU, TBI with AIS >3 | - | PCS | 317 | 44 (22) | 41 (17) | SF-12 | 1y | -HRQoL did not differ between elderly and younger patients  -mortality and poor neurological outcomes increased ≥70y |
| Câmara-Costa  et al [82] | France | 2019 | Children (TBI) | Age <16y, severe TBI (GCS ≤8), admission <6h to PICU | Missing vital signs upon admission, nonaccidental TBI, neurological or psychiatric comorbidities, learning disorders | PCS | 38 | 15.27 (4.45) | 27.5 (9.71) | PedsQL | 7y | -30-50% reported high levels of fatigue  -fatigue levels were associated with overall disability, explaining >60% of the variance in HRQoL |
| Llaquet-Bayo  et al [83] | Spain | 2019 | General | Age ≥16y, treatment in ICU | Death before hospital discharge | PCS | 200 | 47.8 (19.9) | 15.2 (7.9) | EQ-5D-5L | 1y, 2y | -EQ-5D-5L scores showed improvement but did not reach Spanish population norms  -indicator: females and age ≥55y |
| Prieto-Palomino  et al [84] | Spain | 2020 | TBI | Treatment in ICU for TBI in Malaga, Spain | - | PCS | 238 | 35 (22-56) | # | PAEEC | 1y, 3y | - 62.6% reported improved HRQoL |
| García-Rudolph  et al [85] | Spain | 2021 | SCI | Age 14-85y, traumatic SCI, fluent in Spanish | >25y since injury, concomitant brain injury, congenital conditions | RCS | 975 | 34.98 (15.36) | # | HADS, WHOQOL-BREF, TCIQ, CIQ | >10y | -in patients with tetraplegia self-care and transfer abilities predicted home integration  -depression was a key determinant for poorer HRQoL |
| Martino  et al [86] | Italy | 2020 | General | ISS ≥16, treatment in ICU | Non-trauma related disability | CCS | 428 | 39.1 (20.1) | 27 (14) | EQ-5D-NFS, PCL | 10y | - 49.2% had some degree of disability  -the median EQ-5D index was 0.725  -indicators: age, ISS, and GCS ≤8 |
| Sale  et al [87] | Italy | 2012 | SCI | tSCI | Non-tSCI | PCS | 403 | 41.9 (16.2) | # | VAS | 4y | -31% reported satisfaction with sexual life, mainly predicted by bladder function  -driving ability predicted sentimental life satisfaction |
| Ahmed  et al [88] | United Kingdom | 2017 | General | Age ≥18y, ISS ≥9, registration in TARN database | Residency abroad | PCS | 73 | 61 (20.85) | 13.5 (#) | EQ-5D-5L | 1y | - 59% reported mobility issues  -35% experienced personal limitations  -55.9% were fully employed |
| Christie  et al [89] | United Kingdom | 2025 | General | Age ≥16y, admission to critical care in a Trauma Centre within LMTS | - | PCS | 285 | 55 (36-71) | 25 (16-43) | EQ-5D-5L, WHODAS-2 | 1y | -63% had a delirium during critical care, who reported worse HRQoL |
| Kennedy  et al [90] | United Kingdom | 2001 | SCI | Age 16-65y, tSCI | - | PCS | 85 | 32.6 (#) | # | IES, BDI, SAI | 3m | -14% reported trauma-related distress  -indicators: females, anxiety, and depression |
| Hatcher  et al [91] | United Kingdom | 2009 | SCI | Age 18-65y, ≥12w post injury, tSCI, native in English | - | CCS | 102 | 30.39 (11.95) | # | IES, PSS, PANAS, TAS, PTCI | >10y | -high levels of posttraumatic stress were reported  -indicators: negative self-cognitions and neuroticism |
| Bott  et al [92] | United Kingdom | 2019 | Pelvic injury | Age ≥16y, operatively treated unstable pelvic fractures, polytraumatized patients | Untraceable patients | PCS | 74 | 57 (14) | 17 (10) | EQ-5D-3L, SF-36 | >10y | -long-term physical limitations, bodily pain, and vitality were reported |
| Graham  et al [93] | Scotland | 2007 | General | ISS ≥16, ≥2y after trauma | Significant head injury (AIS ≥2) | CCS | 19 | 39 (16-94) | 19 (16-66) | SF-36 | 2y | -the physical and mental health status was poorer compared to population norms |
| Graham  et al [94] | Scotland | 2007 | General | ISS ≥16, GCS 13-15, AIS for head region <2, ≥2y after injury, survival ≥90d | - | RCS | 124 | 35 (16-94) | 19 (16-66) | SF-12, SF-36 | 2y | -the health status was worse compared to population norms |
| Sandström  et al [95] | Sweden | 2020 | General | Age ≥18y, NISS ≥16, fluent in Swedish | - | PCS | 210 | 48.16 (19.1) | 21.25 (12.085) | EQ-5D-3L | 3m, 6m, 1y | -15.8% reported problems in usual activities  -13.2% reported anxiety/depression symptoms  -13.4% reported pain/discomfort |
| Stenberg  et al [96] | Sweden | 2015 | TBI | Age 18-65y, severe TBI (GCS ≤8), requiring ICU admission, fluent in Swedish or Icelandic | Death or expected death ≤3w post injury | PCS | 100 | 42 (17-65) | # | HADS | 1m, 3m, 1y | -cognition improved from 3w to 3m after injury but stabilized afterwards |
| Stålnacke  et al [97] | Sweden | 2019 | TBI | Age 18-65y, acute severe TBI (GCS ≤8) | Death <3w | PCS | 37 | 49 (27-70) | # | HADS | 3m, 1y, 7y | -initially significant improvement in disability and cognitive function was reported  -after 1y no significant changes were reported |
| Norrbrink Budh  et al [98] | Sweden | 2003 | SCI | tSCI | - | PCS | 291 | 47.04 (#) | # | VAS | 1y | -63.7% reported pain, of whom 45.7% reported neurogenic pain |
| Österåker  et al [99] | Sweden | 2005 | SCI | tSCI | Non-tSCI | PCS | 36 | 40 (16-76) | # | BDI | BL, 6m | -25% showed indicators of high alcohol consumption  -many patients exhibited personality traits outside normal ranges |
| Jörgensen  et al [100] | Sweden | 2017 | SCI | Age ≥50y, ≥10y after tSCI | Progressive SCI | CCS | 123 | 63 (9) | # | SWLS, VAS | >10y | -moderate activity limitations were observed, with injury level and spasticity explaining 68% of the variance |
| Horneman  et al [101] | Sweden | 2005 | Children (TBI) | Age <18y, severe TBI | - | PCS | 109 | 10.09 (4.77) | # | QOL-15D | >10y | -HRQoL was significantly worse compared to a matched control group |
| Gross  et al [102] | Switzerland | 2010 | General | ISS ≥16, ≥2 affected body regions | Transferrals | PCS | 115 | 39.5 (20.6) | 27.5 (8.2) | EQ-5D-NFS, SF-36, NHP, SMFA | BL, 2y | -reduced capacity to work was reported in 53% |
| Gross  et al [103] | Switzerland | 2011 | General | ISS ≥16, ≥2 affected body regions, blunt trauma | Transferrals | PCS | 102 | 39.7 (20.5) | 27.1 (8.0) | EQ-5D-NFS, SF-36, NHP, TOP, SMFA | 2y | -persisted pain was reported by 46-85% of patients |
| Attenberger et al [104] | Switzerland | 2011 | General | Polytrauma survivors treated ≥2y previously, ≥2 affected AIS regions, ISS ≥16 | Transferrals | PCS | 117 | 39.6 (20.5) | 27.45 (8.19) | EQ5D-NFS, SF-36, NHP, TOP, SMFA, POLO-chart | 2y | -HRQoL ranged from 52.6 for mental functioning to 79.0 for daily activities  -46% reported abnormal functioning or pain |
| Von Elm  et al [105] | Switzerland | 2008 | TBI | Severe TBI (GCS ≤8), blunt trauma, hospital admission | - | PCS | 27 | 41.1 (1.6-87.9) | # | SF-12 | 6m | -53% experienced significant morbidity |
| Haller  et al [106] | Switzerland | 2015 | TBI | Adults, severe TBI (GCS ≤8), HAIS >3, admission to Swiss trauma centres | Inadequate mental status measured by the NRS-R | PCS | 113 | 48.83 (20.94) | # | SF-12, NEO-FFI, LMS | 6m, 1y | -the German short form of the LMS was validated, resulting in a six-item unidimensional scale with high internal consistency (α = 0.82) and good test-retest reliability (ICC = 0.46) |
| Bosma  et al [107] | Switzerland | 2018 | TBI | Age ≥16y, severe TBI (HAIS >3), resident in the Swiss-speaking cantons in Switzerland, fluent in Swiss, German, French, or English | Unclear trauma history | PCS | 108 | # | # | PCRS-NR, SF-12 | 3m, 6m, 1y | -severity of posttraumatic stress symptoms was significantly affecting cognitive functioning (*p* < 0.001) and mental HRQoL (*p* = 0.01)  -patients 50y showed significant associations with total and emotional functioning |
| Maillard  et al [108] | Switzerland | 2019 | TBI | Age ≥16, severe TBI (HAIS >3) | - | PCS | 116 | 50.8 (25.9-62.8) | 25 (20-29) | QOLIBRI | 4y | -24.1% reported poor HRQoL  -indicators: GCS <13 and working situation |
| Lude  et al [109] | Switzerland | 2014 | SCI | tSCI | - | PCS | 335 | 38 (17.1) | 25 (10.6) | SIP-136 | 1y | -HRQoL improved over the first 2y, especially in the physical domain |
| Overgaard  et al [110] | Denmark | 2011 | General | Age ≥15, ISS ≥9 | Multiple admissions during study period, living abroad, influent in Danish | PCS | 153 | 34 (15-89) | 17 (9-75) | SF-36 | 6-9y | -HRQoL was significantly lower in the poly trauma patients group on all SF-36 domains compared to a matched control group (*p* < 0.001)  -52% returned to work and 20% retired early |
| Wad  et al [111] | Denmark | 2018 | General | Age ≥18y, injury to ≥1 organ system, ≥1 fracture of the long bones or axial skeleton (pelvis or acetabulum), fracture of the axial skeleton in combined with another fracture | Living abroad | PCS | 63 | 32.7 (11.9) | 16 (4-33) | EQ-5D-NFS | >10y | -the EQ-5D scores remained lower than the Danish norm  -indicators: higher ISS |
| Andresen  et al [112] | Denmark | 2016 | SCI | Age ≥18y, tSCI | - | CCS | 537 | 54.6 (14.6) | # | NRS | >10y | -73% reported chronic pain, mainly neuropathic pain (60%)  -71% reported spasticity  -indicators: females, tetraplegia, and shorter time since injury |
| Stergiannis et al [113] | Greece | 2014 | General | Age 14y, treatment at ICU for ≥24h, trauma due to RTA | Severe head injury causing cognitive impairments and communication disability | PCS | 85 | 38.8 (18.1) | # | EQ-5D-NFS | 6m, 1y, 1.5y, 2y | -significant decreases of severe problems were reported in the domains mobility, self-care, and anxiety |
| Papadakaki et al [114] | Greece | 2017 | General | Age ≥18y, multiple injuries due to RTA, treatment in (sub)ICU for ≥1d in Greece, Germany, or Italy | Coma status, deceased during enrolment period | PCS | 93 | 41.8 (16.7) | # | IES, CES-D | BL, 6m, 1y | -significant physical and psychological impacts were observed over time |
| Papadakaki et al [115] | Greece | 2018 | General | Age ≥18y, injury sustained at RTI, acquired admission to (sub)ICU for ≥1d sufficient ability to communicate and understand the research | - | PCS | 54 | # | # | IES, CES-D | 1y | -physical disability increased over the first-year post injury  -PTSD symptoms of avoidance remained high, intrusion symptoms improved |
| Matzaroglou et al [116] | Greece | 2005 | SCI | Age <65y, severe cervical SCI | - | PCS | 39 | 46.7 (#) | # | FSFI | 2y | -s compared to an age and socio-demographically matched control group  -desire, emotional quality of sex life, and overall sex satisfaction were similar between the two groups |
| Kivisild  et al [117] | Estonia | 2014 | SCI | SCI, injured between 2003-2007, treatment in participating rehabilitation and regional hospitals | Influent in Estonian | PCS | 80 | 38.9 (14.8) | # | RAND-36, SSQ, LOS, SAI | 4y | -physical functioning and physical role limitations were the most reported HRQoL deficits, with lower RAND-36 scores compared to other countries |
| Baranyi  et al [118] | Austria | 2008 | General | Age 15-70y, ISS ≥12 | - | RCS | 52 | 37.6 (14.2) | 33.06 (12.61) | SF-36, IES, DBI, DES, SSS, FDS | 1y | - 25% met the diagnostic criteria for PTSD  -21.1% had subsyndromal PTS |
| Byra  et al [119] | Poland | 2020 | SCI | Age ≥18y, tSCI | - | CCS | 163 | 39.6 (9.38) | # | HFS, MALS, ADAPSS | >10y | -forgiveness was significantly correlated with all dimensions of disability acceptance |
| *AED* = Accident and Emergency Department, *AIS* = Abbreviated Injury Scale, *BG* = Berufsgenossenschatlichen Unfallklinik, *BL* = baseline, *CCS* = Case Control Study, *FRI* = Fracture Related Infection, *GCS* = Glasgow Coma Scale, *HAIS* = Head Abbreviated Injury Scale, *HET* = High Energy Trauma, *ICU* = Intensive Care Unit, *ISS* = Injury Severity Scale, *LET* = Low Energy Trauma, *LMTS* = London Major Trauma System, *NISS* = New Injury Severity Scale, *NFS* = Not Further Specified, *OUH* = Oslo University Hospital, *PCS* = Prospective Cohort Study, *PICU* = Pediatric Intensive Care Unit, *RCS* = Retrospective Cohort Study, *RCT* = Randomized Controlled Trial, *RTA* = Road Traffic Accident, *(t)SCI* = (Traumatic) Spinal Cord Injury, *SOC* = Sence Of Coherence, *SRH* = Sunnaas Rehabilitation Hospital, *TARN* =Trauma Audit & Research Network, *TBI* = Traumatic Brain Injury, *TBSA* = Total Body Surface Area, *TR-DGU* = TraumaRegister der Deutscher Gesellschaft für Unfallchirurgie, *UK*= United Kingdom, *QoL* = Quality of Life.  *ADAPSS* = Appraisals of Disability Primary and Secondary Scale, *ADS-K* = Allgemeine Depressionsskala Kurzform, *AEE* = Attitudes to Emotional Expression Scale, *BACQ* = Brief Approach/Avoidance Coping Questionnaire, *BDI* = Beck Depression Index, *BICQ* = Brain Injury Complaint Questionnaire, *BIS/BAS* = Behavioral Inhibition System/Behavioral Activation System, *BSHS-B*= Burn Specific Health Scale-Brief, *CFQ* = Cognitive Failure Questionnaire, *CFS* = Chalder Fatigue Scale, *CES-D* = Center for Epidemiological Studies Depression Scale, *COG* = Self-Assessed Cognitive Functioning scale, *COQ* = Self-Assessed Cognitive Functioning scale, *DES* =Dissociative Experiences Scale, *DEX* = Dysexecutive Questionnaire, *EQ-5D*= EuroQol-5D, *FAI* = Frenchay Activities Index, *FDS* = Fragebogen 24 Dissoziatieven Symptomen, *FQ* = Fatigue Questionnaire, *FSFI* = Female Sexual Function Index, *F-SOZU-K-22* = Social Support Questionnaire, *FSS* = Fatigue Severity Scale, *GARS* = Groningen Activity Restriction Score, *GHQ-20* = 20-item General Health Questionnaire, *GSCL* = Giessen Subjective Complaints List, *HADS* = Hospital Anxiety and Depression Scale, *HASPOC* = Hannover Score for Polytrauma Outcome, *HFS* = Heartland Forgiveness Scale, *HISC* = Head Injury Symptom Checklist, *HUI* = Hospital Utility Index, *ICQ* =Illness Cognition Questionnaire, *IES* = Impact Event Scale, *iMCQ* = iMTA Medical Consumption Questionnaire, *iPCQ* = iMTA Productivity Cost Questionnaire, *LIMB-Q* = Limb Questionnaire, *LMS* = Langer Mindfulness Scale, *LOS* = Life Orientation Scale, *LOT-R* = Life Orientation Test - Revised, *MALS* = Multidimensional Acceptance of Loss Scale, *MFI-20* = Multidimensional Fatigue Inventory, *MHLC* = Multidimensional Health Locus of Control, *MPS* = Majeed Pelvic Score, NEO*-FFI*= Neo-Five Factor Inventory, *NHP* = Nottingham Health Profile, *NRS* = Numeric Rating Scale, *NRS-*R = Neurobehavioral Rating Scale - Revised, PAEEC = Questionnaire for Critically Ill Patients, *PANAS* = Positive and Negative Affect Schedule, *PCL* = The Problem Checklist, *PCRS-NR* = Patient Competency Rating Scale - Neurorehabilitation, *PDS* = Posttraumatic Stress Diagnostic Scale, *PedsQL* = Pediatric Quality of Life, *POLO-chart* = Polytrauma Outcome – Long-term Outcome, *PRWHE* = Patient-Rated Wrist/Hand Evaluation, *PSQI* = Pittsburgh Sleep Quality Index, *PSS*, = Perceived Social Support Scale, *PTCI* = Posttraumatic Cognition Inventory, *PTGI* = Post-Traumatic Growth Inventory, *PTSS-10* = Post-Traumatic Symptom Scale – 10 items, *QDASH* = Quick Disability of Arms/Shoulders/Hand, *QOL-15D* = Quality of Life Instrument-15D, *QOLIBRI* = Quality of Life after Brain Injury, *RAND-36* = Medical Outcome Study 36-Item, *RPQ* = Rivermead Post concussion Symptoms Questionnaire, *RSA* = Resilience Scale for Adults, *SAI* = State Anxiety Inventory, *SDQ* = Strengths and Difficulties Questionnaire, *SF-12* = 12-Item Short Form Health Survey, *SF-36* = 36-Item Short Form Health Survey, *SIP-68* = Sickness Impact Profile-68, *SIP-136* = Sickness Impact Profile-136, *SMFA* = Short Musculoskeletal Function Assessment, *SOC-13* = Sense of Coherence, *SQLP* = Subjective Quality of Life Profile, *SSES-9* = Sherer's Self Efficacy Scale, *SSQ* =Brief Social Support Questionnaire, *SSS* = Social Support Survey, *STAI* = State-Trait Anxiety Inventory, *SWLS* = Satisfaction With Life Scale, *TAS* = Toronto Alexithymia Scale, *TLEQ* = Traumatic Life Events Questionnaire, *TOP* = Trauma Outcome Profile, *UCLA* = UCLA Loneliness Scale, *USER-P* = Utrecht Scale for Evaluation of Rehabilitation-Participation, *VAS* = Visual Analogue Scale, *WHODAS-2* = World Health Organization Disability Assessment Schedule, *WHOQOL-BREF* = World Health Organization Quality of Life Questionnaire.  # = missing value. | | | | | | | | | | | | |

| **Table 2**: Quality assessment of all 119 included studies. | | | | | | | | | | | | | | | |
| --- | --- | --- | --- | --- | --- | --- | --- | --- | --- | --- | --- | --- | --- | --- | --- |
|  | **Reporting incomplete outcome data** | **Clearly described inclusion criteria** | **Data was part of a Trauma Registry** | **Specific or frequently published study cohort** | **Clear definition of severe injury** | **Clearly described response rate** | **Clearly described potential recall bias** | **Used PROMs were clearly described** | **Clearly described physical comorbidities** | **Clearly described psychiatric comorbidities** | **Clearly described route of data collection** | **Clearly described data collecting personnel** | **clearly described who completed the questionnaire** | **Low risk of bias (%)** |  |
| **Ahmed, 2017** |  |  |  |  |  |  |  |  |  |  |  |  |  | 77 |  |
| **Andelic, 2009** |  |  |  |  |  |  |  |  |  |  |  |  |  | 85 |  |
| **Andresen, 2016** |  |  |  |  |  |  |  |  |  |  |  |  |  | 54 |  |
| **Angerpointner, 2021** |  |  |  |  |  |  |  |  |  |  |  |  |  | 77 |  |
| **Anke, 2015** |  |  |  |  |  |  |  |  |  |  |  |  |  | 85 |  |
| **Attenberger, 2012** |  |  |  |  |  |  |  |  |  |  |  |  |  | 62 |  |
| **Azouvi, 2016** |  |  |  |  |  |  |  |  |  |  |  |  |  | 77 |  |
| **Baranyi, 2010** |  |  |  |  |  |  |  |  |  |  |  |  |  | 77 |  |
| **Bosma, 2018** |  |  |  |  |  |  |  |  |  |  |  |  |  | 77 |  |
| **Bott, 2019** |  |  |  |  |  |  |  |  |  |  |  |  |  | 54 |  |
| **Bouman, 2017** |  |  |  |  |  |  |  |  |  |  |  |  |  | 85 |  |
| **Bouzat, 2019** |  |  |  |  |  |  |  |  |  |  |  |  |  | 85 |  |
| **Bruijel, 2025** |  |  |  |  |  |  |  |  |  |  |  |  |  | 77 |  |
| **Byra, 2020** |  |  |  |  |  |  |  |  |  |  |  |  |  | 54 |  |
| **Câmara-Costa, 2020** |  |  |  |  |  |  |  |  |  |  |  |  |  | 85 |  |
| **Chesnel, 2018** |  |  |  |  |  |  |  |  |  |  |  |  |  | 69 |  |
| **Christie, 2025** |  |  |  |  |  |  |  |  |  |  |  |  |  | 85 |  |
| **Falkenberg, 2017** |  |  |  |  |  |  |  |  |  |  |  |  |  | 77 |  |
| **Ferree, 2017** |  |  |  |  |  |  |  |  |  |  |  |  |  | 69 |  |
| **Fleischhacker, 2018** |  |  |  |  |  |  |  |  |  |  |  |  |  | 54 |  |
| **Freigang, 2025** |  |  |  |  |  |  |  |  |  |  |  |  |  | 77 |  |
| **Freigang, 2020** |  |  |  |  |  |  |  |  |  |  |  |  |  | 62 |  |
| **García-Rudolph, 2021** |  |  |  |  |  |  |  |  |  |  |  |  |  | 69 |  |
| **Gojowy, 2019** |  |  |  |  |  |  |  |  |  |  |  |  |  | 46 |  |
| **Graham, 2007** |  |  |  |  |  |  |  |  |  |  |  |  |  | 85 |  |
| **Graham, 2007** |  |  |  |  |  |  |  |  |  |  |  |  |  | 85 |  |
| **Grauwmeijer, 2014** |  |  |  |  |  |  |  |  |  |  |  |  |  | 92 |  |
| **Gross, 2011** |  |  |  |  |  |  |  |  |  |  |  |  |  | 85 |  |
| **Gross, 2010** |  |  |  |  |  |  |  |  |  |  |  |  |  | 85 |  |
| **Haagsma, 2012** |  |  |  |  |  |  |  |  |  |  |  |  |  | 69 |  |
| **Haller, 2015** |  |  |  |  |  |  |  |  |  |  |  |  |  | 62 |  |
| **Hatcher, 2009** |  |  |  |  |  |  |  |  |  |  |  |  |  | 54 |  |
| **Havermans, 2020** |  |  |  |  |  |  |  |  |  |  |  |  |  | 100 |  |
| **Hermans, 2019** |  |  |  |  |  |  |  |  |  |  |  |  |  | 62 |  |
| **Holtslag, 2006** |  |  |  |  |  |  |  |  |  |  |  |  |  | 69 |  |
| **Holtslag, 2007** |  |  |  |  |  |  |  |  |  |  |  |  |  | 77 |  |
| **Holtslag, 2007** |  |  |  |  |  |  |  |  |  |  |  |  |  | 62 |  |
| **Holtslag, 2008** |  |  |  |  |  |  |  |  |  |  |  |  |  | 46 |  |
| **Holtslag, 2007** |  |  |  |  |  |  |  |  |  |  |  |  |  | 69 |  |
| **Horneman, 2005** |  |  |  |  |  |  |  |  |  |  |  |  |  | 54 |  |
| **Jaekel, 2024** |  |  |  |  |  |  |  |  |  |  |  |  |  | 77 |  |
| **Jakimovska, 2017** |  |  |  |  |  |  |  |  |  |  |  |  |  | 54 |  |
| **Janssen, 2008** |  |  |  |  |  |  |  |  |  |  |  |  |  | 46 |  |
| **Janssens, 2009** |  |  |  |  |  |  |  |  |  |  |  |  |  | 62 |  |
| **Janssens, 2009** |  |  |  |  |  |  |  |  |  |  |  |  |  | 69 |  |
| **Jörgensen, 2017** |  |  |  |  |  |  |  |  |  |  |  |  |  | 69 |  |
| **Jourdan, 2016** |  |  |  |  |  |  |  |  |  |  |  |  |  | 69 |  |
| **Jourdan, 2013** |  |  |  |  |  |  |  |  |  |  |  |  |  | 69 |  |
| **Kalbas, 2023** |  |  |  |  |  |  |  |  |  |  |  |  |  | 62 |  |
| **Kaske, 2016** |  |  |  |  |  |  |  |  |  |  |  |  |  | 62 |  |
| **Kaske, 2014** |  |  |  |  |  |  |  |  |  |  |  |  |  | 62 |  |
| **Kennedy, 2001** |  |  |  |  |  |  |  |  |  |  |  |  |  | 77 |  |
| **Kivisild, 2014** |  |  |  |  |  |  |  |  |  |  |  |  |  | 62 |  |
| **Kyösti, 2024** |  |  |  |  |  |  |  |  |  |  |  |  |  | 62 |  |
| **Lefering, 2012** |  |  |  |  |  |  |  |  |  |  |  |  |  | 77 |  |
| **Leijdesdorff, 2018** |  |  |  |  |  |  |  |  |  |  |  |  |  | 77 |  |
| **Lidal, 2013** |  |  |  |  |  |  |  |  |  |  |  |  |  | 77 |  |
| **Lidal, 2008** |  |  |  |  |  |  |  |  |  |  |  |  |  | 62 |  |
| **Llaquet Bayo, 2019** |  |  |  |  |  |  |  |  |  |  |  |  |  | 62 |  |
| **Løke, 2022** |  |  |  |  |  |  |  |  |  |  |  |  |  | 69 |  |
| **Lude, 2014** |  |  |  |  |  |  |  |  |  |  |  |  |  | 54 |  |
| **Mailhan, 2005** |  |  |  |  |  |  |  |  |  |  |  |  |  | 62 |  |
| **Maillard, 2020** |  |  |  |  |  |  |  |  |  |  |  |  |  | 54 |  |
| **Martino, 2020** |  |  |  |  |  |  |  |  |  |  |  |  |  | 54 |  |
| **Matzaroglou, 2005** |  |  |  |  |  |  |  |  |  |  |  |  |  | 46 |  |
| **Nooijen, 2017** |  |  |  |  |  |  |  |  |  |  |  |  |  | 69 |  |
| **Norrbrink Budh, 2003** |  |  |  |  |  |  |  |  |  |  |  |  |  | 46 |  |
| **Österåker, 2005** |  |  |  |  |  |  |  |  |  |  |  |  |  | 62 |  |
| **Overgaard, 2011** |  |  |  |  |  |  |  |  |  |  |  |  |  | 77 |  |
| **Papadakaki, 2017** |  |  |  |  |  |  |  |  |  |  |  |  |  | 77 |  |
| **Papadakaki, 2018** |  |  |  |  |  |  |  |  |  |  |  |  |  | 46 |  |
| **Priente, 2001** |  |  |  |  |  |  |  |  |  |  |  |  |  | 62 |  |
| **Post, 2006** |  |  |  |  |  |  |  |  |  |  |  |  |  | 62 |  |
| **Prieto-Palomino, 2020** |  |  |  |  |  |  |  |  |  |  |  |  |  | 69 |  |
| **Quale, 2010** |  |  |  |  |  |  |  |  |  |  |  |  |  | 69 |  |
| **Quale, 2009** |  |  |  |  |  |  |  |  |  |  |  |  |  | 100 |  |
| **Quintard, 2002** |  |  |  |  |  |  |  |  |  |  |  |  |  | 62 |  |
| **Ringburg, 2011** |  |  |  |  |  |  |  |  |  |  |  |  |  | 85 |  |
| **Ringdal, 2025** |  |  |  |  |  |  |  |  |  |  |  |  |  | 54 |  |
| **Riuttanen, 2024** |  |  |  |  |  |  |  |  |  |  |  |  |  | 92 |  |
| **Sale, 2012** |  |  |  |  |  |  |  |  |  |  |  |  |  | 69 |  |
| **Sandström, 2021** |  |  |  |  |  |  |  |  |  |  |  |  |  | 85 |  |
| **Schönenberg, 2014** |  |  |  |  |  |  |  |  |  |  |  |  |  | 54 |  |
| **Schuckink Kool, 2025** |  |  |  |  |  |  |  |  |  |  |  |  |  | 54 |  |
| **Selj, 2025** |  |  |  |  |  |  |  |  |  |  |  |  |  | 62 |  |
| **Simmel, 2013** |  |  |  |  |  |  |  |  |  |  |  |  |  | 85 |  |
| **Snekkevik, 2003** |  |  |  |  |  |  |  |  |  |  |  |  |  | 62 |  |
| **Soberg, 2012** |  |  |  |  |  |  |  |  |  |  |  |  |  | 77 |  |
| **Soberg, 2015** |  |  |  |  |  |  |  |  |  |  |  |  |  | 77 |  |
| **Soberg, 2010** |  |  |  |  |  |  |  |  |  |  |  |  |  | 69 |  |
| **Soberg, 2007** |  |  |  |  |  |  |  |  |  |  |  |  |  | 77 |  |
| **Soberg, 2007** |  |  |  |  |  |  |  |  |  |  |  |  |  | 69 |  |
| **Soberg, 2013** |  |  |  |  |  |  |  |  |  |  |  |  |  | 62 |  |
| **Stålnacke, 2019** |  |  |  |  |  |  |  |  |  |  |  |  |  | 54 |  |
| **Stalp, 2002** |  |  |  |  |  |  |  |  |  |  |  |  |  | 69 |  |
| **Stenberg, 2015** |  |  |  |  |  |  |  |  |  |  |  |  |  | 69 |  |
| **Stergiannis, 2014** |  |  |  |  |  |  |  |  |  |  |  |  |  | 62 |  |
| **Tøien, 2011** |  | **92** |  |  |  |  |  |  |  |  |  |  |  | 92 |  |
| **Tøien, 2010** |  |  |  |  |  |  |  |  |  |  |  |  |  | 85 |  |
| **Ulvik, 2008** |  |  |  |  |  |  |  |  |  |  |  |  |  | 69 |  |
| **Van Baalen, 2007** |  |  |  |  |  |  |  |  |  |  |  |  |  | 69 |  |
| **Van Delft-Scheurs, 2017** |  |  |  |  |  |  |  |  |  |  |  |  |  | 69 |  |
| **Van Delft-Scheurs, 2014** |  |  |  |  |  |  |  |  |  |  |  |  |  | 92 |  |
| **Van Delft-Scheurs, 2014** |  |  |  |  |  |  |  |  |  |  |  |  |  | 92 |  |
| **Van Delft-Scheurs, 2016** |  |  |  |  |  |  |  |  |  |  |  |  |  | 92 |  |
| **Van der Vlegel, 2021** |  |  |  |  |  |  |  |  |  |  |  |  |  | 69 |  |
| **Van Ditshuizen, 2022** |  |  |  |  |  |  |  |  |  |  |  |  |  | 85 |  |
| **Van Ditshuizen, 2025** |  |  |  |  |  |  |  |  |  |  |  |  |  | 85 |  |
| **Van Ditshuizen, 2023** |  |  |  |  |  |  |  |  |  |  |  |  |  | 85 |  |
| **Van Erp, 2014** |  |  |  |  |  |  |  |  |  |  |  |  |  | 69 |  |
| **Van Leeuwen, 2012** |  |  |  |  |  |  |  |  |  |  |  |  |  | 85 |  |
| **Van Loon, 2011** |  |  |  |  |  |  |  |  |  |  |  |  |  | 54 |  |
| **Vardon-Bounes, 2021** |  |  |  |  |  |  |  |  |  |  |  |  |  | 85 |  |
| **Vassend, 2011** |  |  |  |  |  |  |  |  |  |  |  |  |  | 69 |  |
| **Vles, 2005** |  |  |  |  |  |  |  |  |  |  |  |  |  | 54 |  |
| **Von Elm, 2008** |  |  |  |  |  |  |  |  |  |  |  |  |  | 62 |  |
| **Wad, 2018** |  |  |  |  |  |  |  |  |  |  |  |  |  | 69 |  |
| **Wijnen, 2019** |  |  |  |  |  |  |  |  |  |  |  |  |  | 69 |  |
| **Zwingmann, 2016** |  |  |  |  |  |  |  |  |  |  |  |  |  | 31 |  |
| **Low risk of bias (%)** | 79 | 97 | 52 | 82 | 97 | 92 | 34 | 97 | 36 | 32 | 82 | 54 | 76 |  |  |
| = Low risk of bias  = = High risk of bias | | | | | | | | | | | | | | | |

**Figures**

**Figure 1**: Treemap of all used PROMs across the 119 included studies.

1.	Vles, W.J., et al., *Prevalence and determinants of disabilities and return to work after major trauma.* J Trauma, 2005. **58**(1): p. 126-35.

2.	Post, R.B., C.K. van der Sluis, and H.J. Ten Duis, *Return to work and quality of life in severely injured patients.* Disabil Rehabil, 2006. **28**(22): p. 1399-404.

3.	Holtslag, H., et al., *Long-term outcome after lower extremity injuries in severely injured patients.* European Journal of Trauma, 2006. **32**(4): p. 365-373.

4.	Holtslag, H.R., et al., *Long-term functional health status of severely injured patients.* Injury, 2007. **38**(3): p. 280-9.

5.	Holtslag, H.R., et al., *Determinants of long-term functional consequences after major trauma.* J Trauma, 2007. **62**(4): p. 919-27.

6.	Holtslag, H.R., et al., *Return to work after major trauma.* Clin Rehabil, 2007. **21**(4): p. 373-83.

7.	Holtslag, H.R., et al., *Individual and population burdens of major trauma in the Netherlands.* Bull World Health Organ, 2008. **86**(2): p. 111-7.

8.	Ringburg, A.N., et al., *Prevalence and prognostic factors of disability after major trauma.* J Trauma, 2011. **70**(4): p. 916-22.

9.	Haagsma, J.A., et al., *Prevalence rate, predictors and long-term course of probable posttraumatic stress disorder after major trauma: a prospective cohort study.* BMC Psychiatry, 2012. **12**: p. 236.

10.	van Erp, S., H.R. Holtslag, and E.F. van Beeck, *Determinants of limitations in unpaid work after major trauma: a prospective cohort study with 15 months follow-up.* Injury, 2014. **45**(3): p. 629-34.

11.	van Delft-Schreurs, C.C., et al., *A cross-sectional study of psychological complaints and quality of life in severely injured patients.* Qual Life Res, 2014. **23**(4): p. 1353-62.

12.	van Delft-Schreurs, C.C., et al., *Quality of life in severely injured patients depends on psychosocial factors rather than on severity or type of injury.* Injury, 2014. **45**(1): p. 320-6.

13.	van Delft-Schreurs, C.C., et al., *Psychometric properties of the Dutch Short Musculoskeletal Function Assessment (SMFA) questionnaire in severely injured patients.* Injury, 2016. **47**(9): p. 2034-40.

14.	van Delft-Schreurs, C., et al., *The relationship between physical and psychological complaints and quality of life in severely injured patients.* Injury, 2017. **48**(9): p. 1978-1984.

15.	Ferree, S., et al., *Fractures and dislocations of the hand in polytrauma patients: Incidence, injury pattern and functional outcome.* Injury, 2017. **48**(4): p. 930-935.

16.	Bouman, A.I., et al., *Effects of an Integrated 'Fast Track' Rehabilitation Service for Multi-Trauma Patients: A Non-Randomized Clinical Trial in the Netherlands.* PLoS One, 2017. **12**(1): p. e0170047.

17.	Leijdesdorff, H.A., et al., *Reduced quality of life, fatigue, and societal participation after polytrauma.* International Surgery, 2018. **103**(3-4): p. 158-166.

18.	Wijnen, B.F.M., et al., *Cost-effectiveness of an integrated 'fast track' rehabilitation service for multi-trauma patients: A non-randomized clinical trial in the Netherlands.* PLoS One, 2019. **14**(3): p. e0213980.

19.	Havermans, R.J.M., et al., *Longitudinal analysis of health status the first year after trauma in severely injured patients.* Scand J Trauma Resusc Emerg Med, 2020. **28**(1): p. 29.

20.	van der Vlegel, M., et al., *Long-term medical and productivity costs of severe trauma: Results from a prospective cohort study.* PLoS One, 2021. **16**(6): p. e0252673.

21.	van Ditshuizen, J.C., et al., *Health-related quality of life and return to work 1 year after major trauma from a network perspective.* Eur J Trauma Emerg Surg, 2022. **48**(3): p. 2421-2431.

22.	Van Ditshuizen, J.C., et al., *Comparing health status after major trauma across different levels of trauma care.* Injury, 2023. **54**(3): p. 871-879.

23.	Van Ditshuizen, J.C., et al., *Psychological health status after major trauma across different levels of trauma care: A multicentre secondary analysis.* Injury, 2025. **56**(2): p. 112152.

24.	Schuckink Kool, F.H.N., et al., *Identifying Variables Associated with Long-Term Quality of Life after Severe Lower Limb Trauma Using the LIMB-Q.* Plast Reconstr Surg, 2025. **156**(2): p. 297e-304e.

25.	Van Baalen, B., et al., *Being restricted in participation after a traumatic brain injury is negatively associated by passive coping style of the caregiver.* Brain Inj, 2007. **21**(9): p. 925-31.

26.	Grauwmeijer, E., M.H. Heijenbrok-Kal, and G.M. Ribbers, *Health-related quality of life 3 years after moderate to severe traumatic brain injury: a prospective cohort study.* Arch Phys Med Rehabil, 2014. **95**(7): p. 1268-76.

27.	Bruijel, J., et al., *Course of Fatigue and Sleep After Moderate to Severe Traumatic Brain Injury.* J Head Trauma Rehabil, 2025.

28.	van Leeuwen, C.M., et al., *Relationships between activities, participation, personal factors, mental health, and life satisfaction in persons with spinal cord injury.* Arch Phys Med Rehabil, 2012. **93**(1): p. 82-9.

29.	Hermans, E., et al., *Quality of life after pelvic ring fractures: Long-term outcomes. A multicentre study.* Injury, 2019. **50**(6): p. 1216-1222.

30.	Nooijen, C.F., et al., *A behavioral intervention promoting physical activity in people with subacute spinal cord injury: secondary effects on health, social participation and quality of life.* Clin Rehabil, 2017. **31**(6): p. 772-780.

31.	Janssens, L., et al., *Long-Term Health-Related Quality of Life in Major Pediatric Trauma: A Pilot Study.* Eur J Trauma Emerg Surg, 2009. **35**(4): p. 371-7.

32.	Janssens, L., et al., *Long-term health condition in major pediatric trauma: a pilot study.* J Pediatr Surg, 2009. **44**(8): p. 1591-600.

33.	Snekkevik, H., et al., *Is sense of coherence stable after multiple trauma?* Clin Rehabil, 2003. **17**(4): p. 443-53.

34.	Soberg, H.L., et al., *Return to work after severe multiple injuries: a multidimensional approach on status 1 and 2 years postinjury.* J Trauma, 2007. **62**(2): p. 471-81.

35.	Soberg, H.L., et al., *Long-term multidimensional functional consequences of severe multiple injuries two years after trauma: a prospective longitudinal cohort study.* J Trauma, 2007. **62**(2): p. 461-70.

36.	Ulvik, A., et al., *Quality of life 2-7 years after major trauma.* Acta Anaesthesiol Scand, 2008. **52**(2): p. 195-201.

37.	Quale, A.J., et al., *Severity of injury does not have any impact on posttraumatic stress symptoms in severely injured patients.* Injury, 2009. **40**(5): p. 498-505.

38.	Quale, A.J. and A.K. Schanke, *Resilience in the face of coping with a severe physical injury: a study of trajectories of adjustment in a rehabilitation setting.* Rehabil Psychol, 2010. **55**(1): p. 12-22.

39.	Soberg, H.L., et al., *Mental health and posttraumatic stress symptoms 2 years after severe multiple trauma: self-reported disability and psychosocial functioning.* Arch Phys Med Rehabil, 2010. **91**(3): p. 481-8.

40.	Tøien, K., et al., *Psychological distress after severe trauma: a prospective 1-year follow-up study of a trauma intensive care unit population.* J Trauma, 2010. **69**(6): p. 1552-9.

41.	Tøien, K., et al., *Health related quality of life in trauma patients. Data from a one-year follow up study compared with the general population.* Scand J Trauma Resusc Emerg Med, 2011. **19**: p. 22.

42.	Vassend, O., et al., *Predicting the long-term impact of acquired severe injuries on functional health status: the role of optimism, emotional distress and pain.* Spinal Cord, 2011. **49**(12): p. 1193-7.

43.	Soberg, H.L., et al., *The trajectory of physical and mental health from injury to 5 years after multiple trauma: a prospective, longitudinal cohort study.* Arch Phys Med Rehabil, 2012. **93**(5): p. 765-74.

44.	Soberg, H.L., et al., *Physical and mental health 10 years after multiple trauma: A prospective cohort study.* J Trauma Acute Care Surg, 2015. **78**(3): p. 628-33.

45.	Selj, J.N., et al., *Health-Related Quality of Life and Unmet Healthcare Needs in the First Year Following Moderate-to-Severe Traumatic Injuries-An Observational Study.* J Clin Med, 2025. **14**(12).

46.	Ringdal, K.G., K.T. Holm, and O. Røise, *The Norwegian national trauma registry: development process and essential data insights.* Scand J Trauma Resusc Emerg Med, 2025. **33**(1): p. 78.

47.	Andelic, N., et al., *Functional outcome and health-related quality of life 10 years after moderate-to-severe traumatic brain injury.* Acta Neurol Scand, 2009. **120**(1): p. 16-23.

48.	Soberg, H.L., et al., *Health-related quality of life 12 months after severe traumatic brain injury: a prospective nationwide cohort study.* J Rehabil Med, 2013. **45**(8): p. 785-91.

49.	Anke, A., et al., *Functional Recovery and Life Satisfaction in the First Year After Severe Traumatic Brain Injury: A Prospective Multicenter Study of a Norwegian National Cohort.* J Head Trauma Rehabil, 2015. **30**(4): p. E38-49.

50.	Løke, D., et al., *Impact of Somatic Vulnerability, Psychosocial Robustness and Injury-Related Factors on Fatigue following Traumatic Brain Injury-A Cross-Sectional Study.* J Clin Med, 2022. **11**(6).

51.	Lidal, I.B., et al., *Health-related quality of life in persons with long-standing spinal cord injury.* Spinal Cord, 2008. **46**(11): p. 710-5.

52.	Lidal, I.B., et al., *Fatigue in persons who have lived with spinal cord injury for >20 years.* Spinal Cord, 2013. **51**(2): p. 103-8.

53.	Jakimovska, V.M., et al., *Psychological distress and user experiences with health care provision in persons living with spinal cord injury for more than 20 years.* Spinal Cord, 2017. **55**(9): p. 864-869.

54.	Pirente, N., et al., *[Quality of life of severely injured patients 1 year after trauma. A matched-pair study compared with a healthy control group].* Unfallchirurg, 2001. **104**(1): p. 57-63.

55.	Stalp, M., et al., *Standardized outcome evaluation after blunt multiple injuries by scoring systems: a clinical follow-up investigation 2 years after injury.* J Trauma, 2002. **52**(6): p. 1160-8.

56.	Janssen, C., et al., *Predicting Health-related Quality of Life of Severely Injured Patients: Sociodemographic, Economic, Trauma, and Hospital Stay-related Determinants.* Eur J Trauma Emerg Surg, 2008. **34**(3): p. 277-86.

57.	Lefering, R., et al., *Quality of life after multiple trauma: validation and population norm of the Polytrauma Outcome (POLO) chart.* Eur J Trauma Emerg Surg, 2012. **38**(4): p. 403-15.

58.	Simmel, S., et al., *[Long-term results after multiple trauma with ISS ≥ 25. Outcome and predictors of quality of life].* Chirurg, 2013. **84**(9): p. 771-9.

59.	Kaske, S., et al., *Quality of life two years after severe trauma: a single-centre evaluation.* Injury, 2014. **45 Suppl 3**: p. S100-5.

60.	Kaske, S., et al., *[Pain assessment of severely injured patients 2 years after trauma].* Schmerz, 2016. **30**(3): p. 273-8.

61.	Zwingmann, J., et al., *Lower Health-Related Quality of Life in Polytrauma Patients: Long-Term Follow-Up After Over 5 Years.* Medicine (Baltimore), 2016. **95**(19): p. e3515.

62.	Falkenberg, L., et al., *Long-term outcome in 324 polytrauma patients: what factors are associated with posttraumatic stress disorder and depressive disorder symptoms?* Eur J Med Res, 2017. **22**(1): p. 44.

63.	Fleischhacker, E., et al., *Long-term changes of patient-reported quality of life after major trauma: The importance of the time elapsed after injury.* Injury, 2018. **49**(2): p. 195-202.

64.	Angerpointner, K., et al., *Quality of life after multiple trauma: results from a patient cohort treated in a certified trauma network.* Eur J Trauma Emerg Surg, 2021. **47**(1): p. 121-127.

65.	Freigang, V., et al., *Reduced Recovery Capacity After Major Trauma in the Elderly: Results of a Prospective Multicenter Registry-Based Cohort Study.* J Clin Med, 2020. **9**(8).

66.	Kalbas, Y., et al., *Post-traumatic growth in polytraumatized patients after 20+ years: a long-term follow-up study of 337 patients treated at a level 1 trauma center.* Eur J Trauma Emerg Surg, 2023. **49**(3): p. 1279-1286.

67.	Jaekel, C., et al., *Implementation of health-related quality of life in the German TraumaRegister DGU® - first results of a pilot study.* Health Qual Life Outcomes, 2024. **22**(1): p. 46.

68.	Freigang, V., et al., *Sex-Specific Factors Affecting Quality of Life After Major Trauma: Results of a Prospective Multicenter Registry-Based Cohort Study.* Healthcare (Basel), 2025. **13**(4).

69.	Schönenberg, M., et al., *Depression, posttraumatic stress, and risk factors following spinal cord injury.* Int J Behav Med, 2014. **21**(1): p. 169-76.

70.	Van Loon, P., et al., *Radiological analysis, operative management and functional outcome of open book pelvic lesions: a 13-year cohort study.* Injury, 2011. **42**(10): p. 1012-9.

71.	Gojowy, D., et al., *Early and late-recorded predictors of health-related quality of life of burn patients on long-term follow-up.* Burns, 2019. **45**(6): p. 1300-1310.

72.	Riuttanen, A., et al., *Health-Related Quality of Life in severely injured patients in Finland: an observational cohort study of 325 patients with 1-year follow-up.* Scand J Trauma Resusc Emerg Med, 2024. **32**(1): p. 45.

73.	Kyösti, E., et al., *Long-Term Quality of Life After Pediatric Traumatic Brain Injury Treated in the Intensive Care Unit.* Pediatr Neurol, 2024. **157**: p. 50-56.

74.	Vardon-Bounes, F., et al., *A study of patients' quality of life more than 5 years after trauma: a prospective follow-up.* Health Qual Life Outcomes, 2021. **19**(1): p. 18.

75.	Quintard, B., et al., *[Life satisfaction and psychosocial outcome in severe traumatic brain injuries in Aquitaine].* Ann Readapt Med Phys, 2002. **45**(8): p. 456-65.

76.	Mailhan, L., P. Azouvi, and A. Dazord, *Life satisfaction and disability after severe traumatic brain injury.* Brain Inj, 2005. **19**(4): p. 227-38.

77.	Jourdan, C., et al., *Predictive factors for 1-year outcome of a cohort of patients with severe traumatic brain injury (TBI): results from the PariS-TBI study.* Brain Inj, 2013. **27**(9): p. 1000-7.

78.	Jourdan, C., et al., *A comprehensive picture of 4-year outcome of severe brain injuries. Results from the PariS-TBI study.* Ann Phys Rehabil Med, 2016. **59**(2): p. 100-6.

79.	Azouvi, P., et al., *Disability and health-related quality-of-life 4 years after a severe traumatic brain injury: A structural equation modelling analysis.* Brain Inj, 2016. **30**(13-14): p. 1665-1671.

80.	Chesnel, C., et al., *Self-awareness four years after severe traumatic brain injury: discordance between the patient's and relative's complaints. Results from the PariS-TBI study.* Clin Rehabil, 2018. **32**(5): p. 692-704.

81.	Bouzat, P., et al., *Modeling the Influence of Age on Neurological Outcome and Quality of Life One Year after Traumatic Brain Injury: A Prospective Multi-Center Cohort Study.* J Neurotrauma, 2019. **36**(17): p. 2506-2512.

82.	Câmara-Costa, H., et al., *Self- and Parent-Reported Fatigue 7 Years After Severe Childhood Traumatic Brain Injury: Results of the Traumatisme Grave de l'Enfant Prospective Longitudinal Study.* J Head Trauma Rehabil, 2020. **35**(2): p. 104-116.

83.	Llaquet Bayo, H., et al., *Analysis of quality of life after major trauma: a spanish follow-up cohort study.* Eur J Trauma Emerg Surg, 2019. **45**(2): p. 289-297.

84.	Prieto-Palomino, M.A., et al., *Improvement of quality of life (QOL) between 1 and 3-4 years after traumatic brain injury (TBI) in ICU patients.* Acta Neurochir (Wien), 2020. **162**(7): p. 1619-1628.

85.	García-Rudolph, A., et al., *Relationships Between Functionality, Depression, and Anxiety With Community Integration and Quality of Life in Chronic Traumatic Spinal Cord Injury.* Am J Phys Med Rehabil, 2021. **100**(9): p. 840-850.

86.	Martino, C., et al., *Long-term outcomes in major trauma patients and correlations with the acute phase.* World J Emerg Surg, 2020. **15**: p. 6.

87.	Sale, P., et al., *Predictors of changes in sentimental and sexual life after traumatic spinal cord injury.* Arch Phys Med Rehabil, 2012. **93**(11): p. 1944-9.

88.	Ahmed, W., R. Alwe, and D. Wade, *One-year functional outcomes following major trauma: experience of a UK level 1 major trauma centre.* Clin Rehabil, 2017. **31**(12): p. 1646-1652.

89.	Christie, R. and E. Cole, *Delirium after major trauma critical care and the association with recovery at 12 months.* Trauma, 2025. **27**(4): p. 307-316.

90.	Kennedy, P. and M.J. Evans, *Evaluation of post traumatic distress in the first 6 months following SCI.* Spinal Cord, 2001. **39**(7): p. 381-6.

91.	Hatcher, M.B., C. Whitaker, and A. Karl, *What predicts post-traumatic stress following spinal cord injury?* Br J Health Psychol, 2009. **14**(Pt 3): p. 541-61.

92.	Bott, A., et al., *Long-Term Patient-Reported Functional Outcome of Polytraumatized Patients With Operatively Treated Pelvic Fractures.* J Orthop Trauma, 2019. **33**(2): p. 64-70.

93.	Graham, C.A., et al., *Long-term outcomes of major trauma without head injury in the West of Scotland: pilot case-control study.* Eur J Emerg Med, 2007. **14**(1): p. 35-8.

94.	Graham, C., N. Craig, and M. Gordon, *Health Status following major trauma in the West of Scotland: pilot descriptive study.* Hong Kong Journal of Emergency Medicine, 2007. **14**(1): p. 29-36.

95.	Sandström, L., et al., *Trauma patients' health-related quality of life and perceptions of care: A longitudinal study based on data from the Swedish Trauma Registry.* Int Emerg Nurs, 2021. **55**: p. 100850.

96.	Stenberg, M., et al., *Cognitive Impairment after Severe Traumatic Brain Injury, Clinical Course and Impact on Outcome: A Swedish-Icelandic Study.* Behav Neurol, 2015. **2015**: p. 680308.

97.	Stålnacke, B.M., B.I. Saveman, and M. Stenberg, *Long-Term Follow-Up of Disability, Cognitive, and Emotional Impairments after Severe Traumatic Brain Injury.* Behav Neurol, 2019. **2019**: p. 9216931.

98.	Norrbrink Budh, C., et al., *Pain in a Swedish spinal cord injury population.* Clin Rehabil, 2003. **17**(6): p. 685-90.

99.	Osteråker, A.L. and R. Levi, *Indicators of psychological distress in postacute spinal cord injured individuals.* Spinal Cord, 2005. **43**(4): p. 223-9.

100.	Jörgensen, S., S. Iwarsson, and J. Lexell, *Secondary Health Conditions, Activity Limitations, and Life Satisfaction in Older Adults With Long-Term Spinal Cord Injury.* Pm r, 2017. **9**(4): p. 356-366.

101.	Horneman, G., et al., *Health-related quality of life of adolescents and young adults 10 years after serious traumatic brain injury.* Int J Rehabil Res, 2005. **28**(3): p. 245-9.

102.	Gross, T., et al., *Factors associated with reduced longer-term capacity to work in patients after polytrauma: a Swiss trauma center experience.* J Am Coll Surg, 2010. **211**(1): p. 81-91.

103.	Gross, T. and F. Amsler, *Prevalence and incidence of longer term pain in survivors of polytrauma.* Surgery, 2011. **150**(5): p. 985-95.

104.	Attenberger, C., F. Amsler, and T. Gross, *Clinical evaluation of the Trauma Outcome Profile (TOP) in the longer-term follow-up of polytrauma patients.* Injury, 2012. **43**(9): p. 1566-74.

105.	von Elm, E., et al., *Severe traumatic brain injury in Switzerland - feasibility and first results of a cohort study.* Swiss Med Wkly, 2008. **138**(23-24): p. 327-34.

106.	Haller, C.S., *Mindful Creativity Scale (MCS): Validation of a German version of the Langer Mindfulness Scale with patients with severe TBI and controls.* Brain Inj, 2015. **29**(4): p. 517-26.

107.	Bosma, C.M., N. Mansoor, and C.S. Haller, *Association of Posttraumatic Stress Symptom Severity With Health-Related Quality of Life and Self-Reported Functioning Across 12 Months After Severe Traumatic Brain Injury.* Arch Phys Med Rehabil, 2018. **99**(8): p. 1576-1583.

108.	Maillard, J., et al., *Prediction of long-term quality of life after severe traumatic brain injury based on variables at hospital admission.* Brain Inj, 2020. **34**(2): p. 203-212.

109.	Lude, P., et al., *Quality of life in and after spinal cord injury rehabilitation: a longitudinal multicenter study.* Top Spinal Cord Inj Rehabil, 2014. **20**(3): p. 197-207.

110.	Overgaard, M., C.B. Høyer, and E.F. Christensen, *Long-term survival and health-related quality of life 6 to 9 years after trauma.* J Trauma, 2011. **71**(2): p. 435-41.

111.	Wad, M.S., et al., *Survival and health related quality of life after severe trauma - a 15 years follow up study.* Injury, 2018. **49**(2): p. 191-194.

112.	Andresen, S.R., et al., *Pain, spasticity and quality of life in individuals with traumatic spinal cord injury in Denmark.* Spinal Cord, 2016. **54**(11): p. 973-979.

113.	Stergiannis, P., et al., *Health-related quality of life and rehabilitation cost following intensive care unit stay in multiple trauma patients.* J Trauma Nurs, 2014. **21**(3): p. 115-21.

114.	Papadakaki, M., et al., *Psychological distress and physical disability in patients sustaining severe injuries in road traffic crashes: Results from a one-year cohort study from three European countries.* Injury, 2017. **48**(2): p. 297-306.

115.	Papadakaki, M., et al., *Physical, psychological and economic burden of two-wheel users after a road traffic injury: Evidence from intensive care units of three EU countries.* J Safety Res, 2018. **67**: p. 155-163.

116.	Matzaroglou, C., et al., *Sexual function in females with severe cervical spinal cord injuries: a controlled study with the Female Sexual Function Index.* Int J Rehabil Res, 2005. **28**(4): p. 375-7.

117.	Kivisild, A., et al., *Health-related quality of life in patients with traumatic spinal cord injury in Estonia.* Spinal Cord, 2014. **52**(7): p. 570-5.

118.	Baranyi, A., et al., *Relationship between posttraumatic stress disorder, quality of life, social support, and affective and dissociative status in severely injured accident victims 12 months after trauma.* Psychosomatics, 2010. **51**(3): p. 237-47.

119.	Byra, S., J. Mróz, and K. Kaleta, *Forgiveness and acceptance of disability in people with traumatic spinal cord injury-the mediating role of disability appraisal. A cross-sectional study.* Spinal Cord, 2020. **58**(12): p. 1317-1324.
